# Upgrading of efficient and scalable CRISPR–Cas-mediated technology for genetic engineering in thermophilic fungus *Myceliophthora thermophila*

**DOI:** 10.1186/s13068-019-1637-y

**Published:** 2019-12-23

**Authors:** Qian Liu, Yongli Zhang, Fangya Li, Jingen Li, Wenliang Sun, Chaoguang Tian

**Affiliations:** 1 Key Laboratory of Systems Microbial Biotechnology, Tianjin Institute of Industrial Biotechnology, Chinese Academy of Sciences, Tianjin, 300308 China; 2 University of Chinese Academy of Sciences, Beijing, 100049 China

**Keywords:** CRISPR–Cas12a, CRISPR–Cas9, Genome editing, *Myceliophthora thermophila*, Marker recycling, Cellulase

## Abstract

**Background:**

Thermophilic filamentous fungus *Myceliophthora thermophila* has great capacity for biomass degradation and is an attractive system for direct production of enzymes and chemicals from plant biomass. Its industrial importance inspired us to develop genome editing tools to speed up the genetic engineering of this fungus. First-generation CRISPR–Cas9 technology was developed in 2017 and, since then, some progress has been made in thermophilic fungi genetic engineering, but a number of limitations remain. They include the need for complex independent expression cassettes for targeting multiplex genomic loci and the limited number of available selectable marker genes.

**Results:**

In this study, we developed an *Acidaminococcus* sp. Cas12a-based CRISPR system for efficient multiplex genome editing, using a single-array approach in *M. thermophila*. These CRISPR–Cas12a cassettes worked well for simultaneous multiple gene deletions/insertions. We also developed a new simple approach for marker recycling that relied on the novel cleavage activity of the CRISPR–Cas12a system to make DNA breaks in selected markers. We demonstrated its performance by targeting nine genes involved in the cellulase production pathway in *M. thermophila* via three transformation rounds, using two selectable markers *neo* and *bar*. We obtained the nonuple mutant M9 in which protein productivity and lignocellulase activity were 9.0- and 18.5-fold higher than in the wild type. We conducted a parallel investigation using our transient CRISPR–Cas9 system and found the two technologies were complementary. Together we called them CRISPR–Cas-assisted marker recycling technology (Camr technology).

**Conclusions:**

Our study described new approaches (Camr technology) that allow easy and efficient marker recycling and iterative stacking of traits in the same thermophilic fungus strain either, using the newly established CRISPR–Cas12a system or the established CRISPR–Cas9 system. This Camr technology will be a versatile and efficient tool for engineering, theoretically, an unlimited number of genes in fungi. We expect this advance to accelerate biotechnology-oriented engineering processes in fungi.

## Background

Filamentous fungi are important decomposers that contribute plant biomass to the biological carbon cycle [1, 2]. The natural ability of these microorganisms to secrete enzymes, organic acids, and secondary metabolites has been harnessed for high-level protein production in biotechnology, food, textile, and pharmaceutical industries [3–5]. Genetic engineering can be a powerful approach for filamentous fungi not only to gain deep elucidation of gene function, but to also improve production levels and minimize unwanted by-product formation [6, 7]. However, the typical efficiency of homologous integration is very low using classical genetic approaches. CRISPR–Cas systems have recently enabled a wide range of applications for genome editing in many organisms [8–12]. Remarkably, and in just the past few years, the CRISPR–Cas9 system has emerged as a significantly efficient strategy to solve the problem of low gene editing frequency in filamentous fungi [13–24]. Most CRISPR–Cas9 systems use plasmids or autonomous replicating vectors to introduce Cas9 and single-guide RNA (sgRNA) into fungal genomes [13–23], although some researchers have successfully used purified RNA/Cas9 protein complexes [24, 25]. The Cas9–sgRNA complex binds to the corresponding target site of the protospacer in a genome and specifically induces double-strand breaks. These breaks can be used as a basis for site-specific mutagenesis mediated by non-homologous end-joining or for the introduction of precise mutation or integration via homology-directed repair.

The other restrictive factor for genetic and metabolic engineering in filamentous fungi arises from the limited number of dominant selectable markers, including the low number of antibiotic resistance and auxotrophic genes. Therefore, it is necessary to identify markers for recycling systems for the introduction of multiple expression constructs or sequential gene deletions. Marker recycling systems have been developed in some filamentous fungi by excision of the marker *pyrG*/*amdS* by counter selection with 5-fluoroorotic acid/5-fluoroacetamide [26–28] or site-specific recombination systems, such as Cre-*lox*P [29–33] and FLP/*FRT* [34, 35], in which the expression of Cre recombinase or flippase (FLP) eliminated the marker cassette flanked by *loxP* or *FRT* sites. The counter-selection method requires auxotrophic strains that can be time-consuming and laborious to build, and therefore the Cre-*lox*P recombination system has been more widely adapted for marker rescue in fungi, such as *Aspergillus nidulans*, *Neurospora crassa*, *Neotyphodium* sp., *Aspergillus oryzae*, *Cryphonectria parasitica*, *Metarhizium robertsii*, *Penicillium oxalicum*, and *Fusarium graminearum* [29–33]. Recently, Katayama et al. [36] established an efficient multiple genetic engineering technique in *A. oryzae* that was based on the CRISPR–Cas9 system and recycling of an AMA1-based plasmid harboring the drug-resistance marker *ptrA*, allowing for repeatable genetic manipulation. More recently, Leynaud-Kieffer et al. [37] reported a simple Cas9-based gene targeting method that provided selectable, iterative, and marker-free generation of genomic editing using the auxotrophic marker *pyrG*.

We previously developed a CRISPR–Cas9 system that efficiently edited the thermophilic filamentous fungi *Myceliophthora thermophila* (Synonym: *Thermothelomyces thermophilus*) and *Myceliophthora heterothallica*, enabling simultaneous multigene disruptions of up to four loci using selectable markers *bar* and *neo* [20]. *M. thermophila* is a potential reservoir of novel industrial thermostable enzymes and has the exceptional potential to produce proteins, chemicals, and biofuels directly from renewable biomass [38–42]. Multiple mutants of *M. thermophila* with double-, triple- and quadruple-deletions and significantly increased cellulase production were generated using this CRISPR–Cas9 genome-engineering tool. However, stacking additional traits to improve lignocellulase production in such mutants was limited, because only one selectable marker (*hph*, conferring hygromycin resistance) remained after the markers *bar* and *neo* were used to generate the primary multiplex-locus deletions. Further, using *hph* might produce a dead-end strain in which no further selective markers are available for additional genetic manipulation. This limitation was ameliorated in this study by implementing an approach that allowed for marker recycling.

**Fig. 1:**
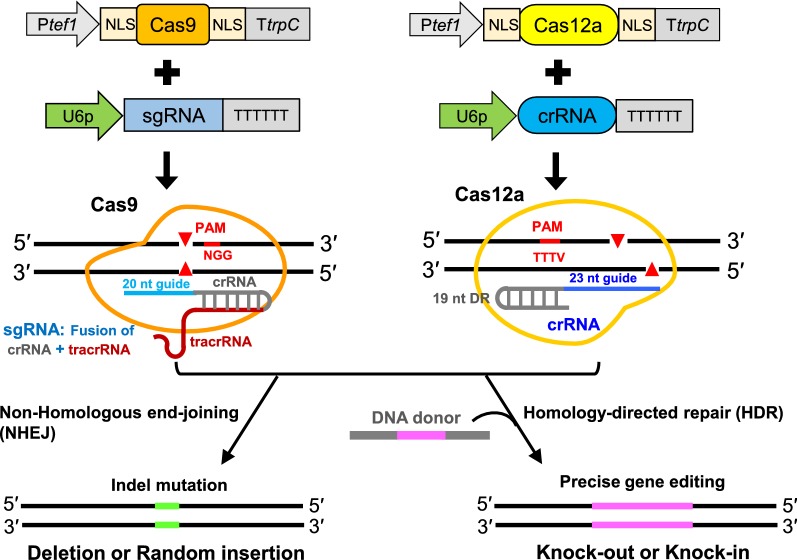
Schematic overview of CRISPR–Cas12a- and CRISPR–Cas9-mediated genome editing in the thermophilic fungus *M. thermophila*. To edit the *M. thermophila* genome, either the Cas9 and sgRNA-expressing cassettes or Cas12a and crRNA expression cassettes were introduced into the recipient protoplasts. For the CRISPR–Cas9 system, the single-guide RNA (sgRNA) contains crRNA and tracrRNA. As compared to Cas9, Cas12a is a single-RNA-guided nuclease that does not require a tracrRNA and the crRNA consists of 19-nt direct repeat and 23-nt guide. Cas12a has a T-rich PAM sequence located at the 5′ end of the protospacer and cleaves DNA distal from the PAM and generates staggered ends. Once Cas9 or Cas12a nuclease generates the sequence-specific double-stranded DNA break (DSB), non-homologous end-joining (NHEJ) or homology-directed repair (HDR) processes mediate DNA modification at the cleavage locus. NHEJ produced small random insertion or deletion (indels) cleaved site, whereas HDR uses DNA donor template for the precise recombination. DR, 19 nt direct repeat; U6p, MtU6 promoter; Ptef1, *Tef1* promoter; NLS, nuclear localization signal; TtrpC, *trpC* Terminator. TTTTTT, poly-T sequence used as terminator

Recently, Cas12a (also known as Cpf1), a member of the class 2 type V-A CRISPR system family, uses a single RuvC catalytic domain for guide RNA double-stranded DNA cleavage and has been harnessed for genome editing [43–45]. Distinct from Cas9, Cas12a possesses several unique and attractive features [9–11] and provides for substantial expansion of the genomic editing toolbox in some eukaryotic organisms [43–51] and bacteria [52–55]. For instance, Cas12a is a single-RNA-guided nuclease that does not need a trans-activating CRISPR RNA (crRNA). Cas12a enzymes mature the CRISPR–RNA array itself without additional RNase [43], recognize a T-rich protospacer adjacent motif (PAM) [44], and generate staggered ends in its PAM-distal target site [44]. Compared to Cas9, one major advantage of Cas12a is its ability to encode two or more crRNAs in a multiplex single transcript by using customized CRISPR arrays [45]. These features generated interest in Cas12a as an expanded minimalistic CRISPR–Cas system for convenient multiplex genes editing and regulation by incorporating multiple crRNAs insulated by short direct repeats [45, 50, 51, 54, 55]. Among the Cas12a (Cpf1) orthologs, three (*Francisella novicida* U112 FnCpf1, *Acidaminococcus* sp. BV3L6 AsCpf1, and *Lachnospiraceae bacterium* ND2006 LbCpf1) have been studied the most both in vivo [45–55] and in vitro [43, 44, 56]. The crRNA used with Cas12a orthologs typically composed of a 23–25 nt guide sequence and a 19 nt direct repeat [43–50]. Thus far, the CRISPR–Cas12a system has been developed only in *A. nidulans* and *A. niger* [57], and *T. thermophilus* [58] as far as we know in filamentous fungi. To test whether the Cas12a effector can be used as an attractive alternative genome editing tool in thermophilic filamentous fungi, in this study, we firstly developed a new efficient CRISPR–Cas12a (AsCpf1) system in *M. thermophila*. Secondly, we established a marker recycling approach based on transient introduction of the new CRISPR–Cas12a system or our previous CRISPR–Cas9 system in *M. thermophila* (Fig. 1). Thirdly, we generated 11 gene modifications in the wild-type strain through three rounds of manipulations as proof-of-concept, which resulted in hyper-cellulase production. We showed that the CRISPR–Cas (Cas12a or Cas9) system is a versatile technology that can rapidly and conveniently generate multi-trait strains, in which iterative stacking of industrially relevant traits is not limited by the selectable marker availability.

## Results

### Construction of CRISPR–Cas12a system in *M. thermophila*

Two Cas12a (Cpf1) orthologs (AsCpf1 and LbCpf1) were reported to have robust DNA cleavage activity in higher eukaryotes with a 5′-TTTV-3′ PAM, where V can be A, C, or G [43]. To investigate its potential editing activity in filamentous fungi, we designed and developed a Cas12a-mediated genome editing tool in *M. thermophila*. Previously, we successfully harnessed the *tef1* promoter P*tef1* and U6 snRNA promoter U6p to drive the expression of Cas9 and sgRNAs, respectively [20]. Thus, in this study, the codon-optimized *Cas12a* (*AsCpf1*) gene and the corresponding crRNA were expressed under the control of P*tef1* and U6p, respectively (Additional file 1: Fig. S1). The CRISPR–Cas12a system consisted of separate *Cas12a* and crRNA expression cassettes (Fig. 1). Briefly, the PCR products of P*tef1*-*Cas12a*-TtrpC and U6p-crRNA were mixed with or without the homology template and co-transformed into *M. thermophila* protoplasts.

### CRISPR–Cas12a-mediated genetic mutation by non-homologous end-joining

**Fig. 2:**
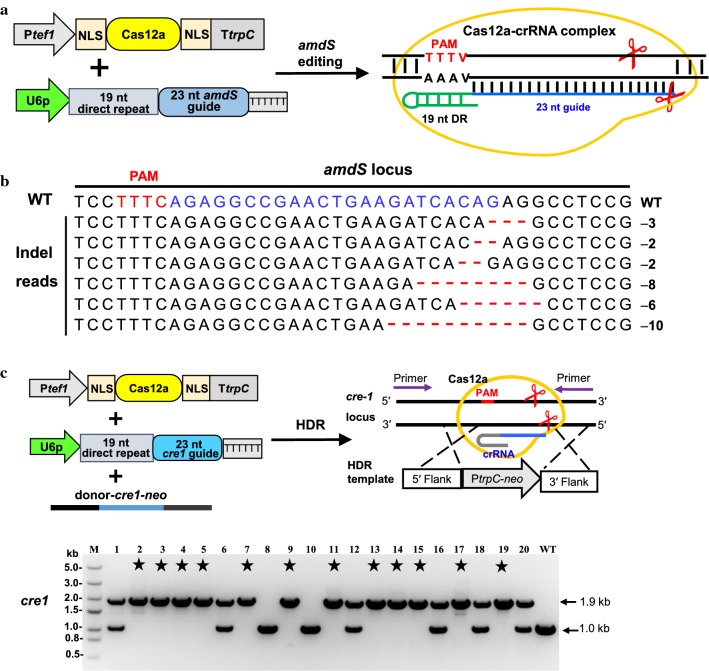
CRISPR–Cas12a system as a new robust genome editing tool in *M. thermophila*. **a** Schematic illustration of the mutagenesis of *amdS* for measuring Cas12a-mediated DNA cleavage in *M. thermophila*. Positive transformants were selected on plates containing 2 mg mL^−1^ FAA. **b** Indel patterns at the *amdS* target locus of FAA-resistant transformants. The number on the right of each sequence is the indel length (−, deletion). Blue, crRNA base-pairing site; red, PAM sequences; WT, wild-type sequence. **c** Schematic illustration of CRISPR–Cas12a-donor DNA-mediated deletion of the target gene *cre*-*1* based on homology-directed repair (HDR). Twenty transformants were selected and verified by PCR analysis. The expected length of the deletion mutant was 1.9 kb, whereas that of the wild-type strain (WT), which was the negative control, was 1.0 kb (rightmost lane). Heterokaryotic transformants showed two PCR bands (both of wild-type and knockout). The symbol of star indicated deletion mutant

The site-specific DNA cleavage created by Cas12a allowed the creation of frameshift insertion and deletion (indel) mutations by non-homologous end-joining repair, which led to loss-of-function of the target genes. To easily determine whether Cas12a was functional in *M. thermophila*, we designed a crRNA expression cassette to target *amdS*, a gene that is essential for growth on acetamide as the only nitrogen source, deletion of which resulted in resistance to fluoroacetamide (FAA). This allowed us to deliver the PCR products of the P*tef1*-*Cas12a*-TtrpC and U6p-*amdS*-crRNA cassettes into protoplasts of the recipient *M. thermophila* strain M1 [20], which contained *amdS* and was sensitive to FAA (Fig. 2a). FAA-resistant transformants were obtained and mutations in *amdS* were verified by DNA sequencing. We obtained six Cas12a-induced indel mutations at sites distal from the PAM and these frameshift mutations were the result of deletions of several nucleotides (Fig. 2b). In a control experiment in which transformation was performed using the Cas12a expressing cassette only, no colonies were obtained on FAA. Together these results demonstrate that delivery of transient PCR products of Cas12a and crRNA cassettes can efficiently mediate mutation of the target gene via non-homologous end-joining repair in *M. thermophila*.

### CRISPR–Cas12a-mediated single gene editing by homology-directed repair (HDR)

Gene editing by HDR is an efficient strategy for precise deletion and insertion in a desired locus. To further evaluate the potential of Cas12a-assisted homology-mediated mutagenesis, we used Cas12a to facilitate loss-of-function of *cre*-*1* (carbon catabolite repression transcription factor) using a DNA repair template. The PCR products of P*tef1*-*Cas12a*-TtrpC, U6p-*cre1*-crRNA, and donor-*cre1*-*neo* were introduced simultaneously into protoplasts of wild-type *M. thermophila*. Then, 20 transformants were picked randomly by G418 selection to determine the gene editing efficiency by PCR analysis using specific primer sets (Fig. 2c). The homologous recombination (HR) efficiencies of gene replacement (including both homokaryons and heterokaryons) for all tested transformants are summarized in Table 1. Compared with transformation with donor DNA alone (15%), the HR frequency of gene replacement (12 homokaryons + 6 heterokaryons) was as high as 90% after co-transformation using the CRISPR–Cas12a system and HR donor DNA (Table 1; Fig. 2c; and Additional file 2: Fig. S1). The HR rates were also low (15% or 10%) when Cas12a or crRNA alone with donor DNA was used for the transformation (Table 1; Additional file 2: Fig. S1). As shown in Fig. 2c, 12 out of 20 (60% deletion efficiency) transformants were confirmed as positive mutant with *cre*-*1* successfully deleted, whereas the control transformation with donor DNA alone or with Cas12a or crRNA alone led to no final correct deletion (0 out 20, 0% deletion efficiency) but only 2 or 3 heterokaryotic mutants of *cre*-*1* gene (Table 1; Additional file 2: Fig. S1). Six out of 20 transformants displayed the heterokaryotic phenotype in our PCR analysis, which showed two bands of correct integration band and wild-type band (Fig. 2c). Based on the multinucleate nature of filamentous fungi, fungal protoplasts contained an uncertain number of nuclei, there are difficulties producing homokaryotic transformants from multinucleate tissue and most of transformants were often heterokaryons through classic method. Streaking conidia may serve to achieve the separation, using sorbose and selective media, and confirming purity by repeated streaking [59]. Cumulatively, these results indicate that similar to the previous *M. thermophila* CRISPR–Cas9 system, the *Acidaminococcus* sp. CRISPR–Cas12a system was very efficient in promoting homologous recombination-mediated gene editing and generating homokaryotic deleted mutants.

**Table 1:** Summary of the genomic editing in *M. thermophila* using the CRISPR–Cas system

Host strain^a^	Target locus	Elements in co-transformation	No. of analyzed transformants	No. of HR transformants^b^	HR efficiency (%)^c^	Each gene disruption efficiency (%)
WT	*cre*-*1*	Donor-*cre1*-*neo*	20	3	15	0
WT	*cre*-*1*	Cas12a +donor-*cre1*-*neo*	20	3	15	0
WT	*cre*-*1*	crRNA +donor-*cre1*-*neo*	20	2	10	0
WT	*cre*-*1*	Cas12a + crRNA + donor-*cre1*-*neo*	20	18	90	60
WT	*cre*-*1*, *res*-*1*, *gh1*-*1*	Donor DNA of *cre*-*1*, *res*-*1* and *gh1*-*1*	23	0	0	0 0 0
WT	*cre*-*1*, *res*-*1*, *gh1*-*1*	Cas12a + pooled three sets of crRNA + donor DNA of *cre*-*1*, *res*-*1* and *gh1*-*1*	20	8	40	35 25 25
WT	*cre*-*1*, *res*-*1*, *gh1*-*1*	Cas12a + array1 + donor DNA of *cre*-*1*, *res*-*1* and *gh1*-*1*	22	7	32	41 32 27
WT	*cre*-*1*, *res*-*1*, *gh1*-*1*	Cas9 + three sets of sgRNA + donor DNA of *cre*-*1*, *res*-*1* and *gh1*-*1*	23	9	39	39 35 39
M3	*neo*,*alp*-*1*, *rca*-*1*, *hcr*-*1*	Donor DNA of *neo*, *alp*-*1*, *rca*-*1* and *hcr1*	23	0	0	0 0 0 0
M3	*neo*,*alp*-*1*, *rca*-*1*, *hcr* -*1*	Cas12a + array2 + donor DNA of *neo*, *alp*-*1*, *rca*-*1* and *hcr*-*1*	23	5	22	26 30 13 22
M3	*neo*, *alp*-*1*, *rca*-*1*, *hcr*-*1*	Cas9 + four sets of sgRNA + donor DNA of *neo*, *alp*-*1*, *rca*-*1* and *hcr*-*1*	23	5	22	35 30 13 30
M7	*bar*, *ap*-*3*, *prk*-*6*	Donor DNA of *bar*, *ap*-*3* and *prk*-*6*	22	0	0	0 0 0
M7	*bar*, *ap*-*3*, *prk*-*6*	Cas12a + array3 + donor DNA of *bar*, *ap*-*3* and *prk*-*6*	22	9	41	32 27 23
M7	*Bar*, *ap*-*3*, *prk*-*6*	Cas9 + three sets of sgRNA + donor DNA of *bar*, *ap*-*3* and *prk*-*6*	21	8	38	33 29 19

^a^WT, wild-type strain; M3, triple-mutant Δ*cre1*Δ*res1*Δ*gh1*-*1*; M7, septuple mutant ∆*cre1*Δ*res1*Δ*gh1*-*1*Δ*neo*Δ*alp1*Δ*rca1*::*xyr1*Δ*hcr1*

^b^HR, homologous recombination

^c^HR efficiency, HR frequency of gene replacement (including both homokaryons and heterokaryon)

### CRISPR–Cas12a-mediated multiplex genome editing using both the pooled crRNA cassettes and a single crRNA array

**Fig. 3:**
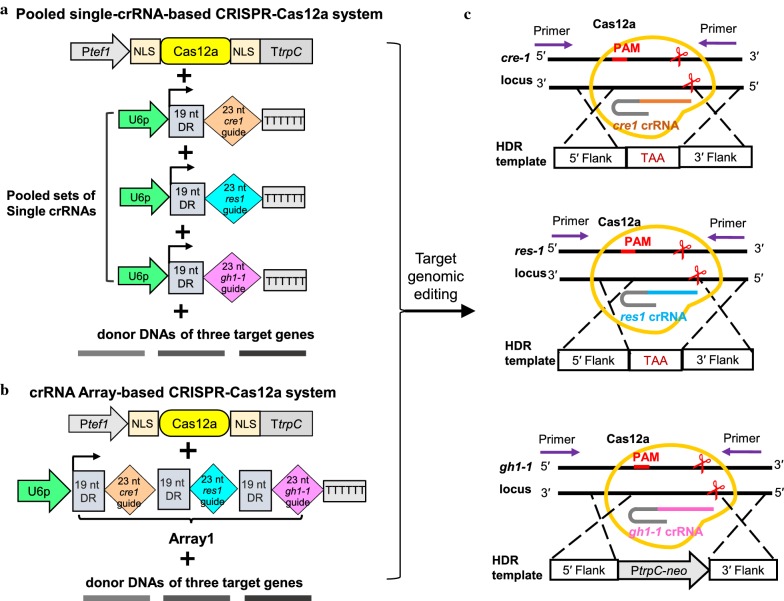
CRISPR–Cas12a-mediated multiplex genome editing in *M. thermophila*. **a** Schematic illustration of the simultaneous deletion of three target genes, *cre*-*1*, *res*-*1* and *gh1*-*1*, using the CRISPR–Cas12a system with pooled single-crRNA cassettes. **b** Schematic illustration of the simultaneous deletion of three target genes, *cre*-*1*, *res*-*1* and *gh1*-*1*, using the CRISPR–Cas12a system with crRNA array1 expressing cassette in the order *cre1*–*res1*–*gh1*-*1*. **c** Once Cas12a nuclease introduces the sequence-specific double-stranded DNA break, homology-directed repair (HDR) mediate precise gene deletion using donor DNA template at the cleavage locus of *cre*-*1*, *res*-*1* and *gh1*-*1*, respectively. DR, 19 nt direct repeat; U6p, MtU6 promoter; Ptef1, *Tef1* promoter; NLS, nuclear localization signal; TtrpC, *trpC* Terminator

To test whether this CRISPR–Cas12a system could efficiently target multiplex genes in *M. thermophila*, we explored its ability to target three different loci simultaneously. Besides *cre*-*1*, two other genes (*res*-*1* and *gh1*-1) involved in cellulase production were chosen as targets. Genome editing using HDR is well suited to modifying genes without introducing unwanted changes. Two markerless donor DNA sequences (donor-*cre1*-TAA and donor-*res*-*1*-TAA) were designed as repair templates for precise seamless gene deletion by introducing stop codon (TAA) centrally in the flank sequence, as well as the donor-*gh1*-*1*-*neo* construct to target *gh1*-*1* to allow G418 selection of transformants (Fig. 3a). Unlike Cas9, Cas12a required only one short 42 nt crRNA composed of 19 nt direct repeat and 23 nt guide, so no trans-activating crRNA was needed. Distinct from Cas9, one major advantage of Cpf1 possesses the ability to process a customized crRNA array both in vitro and in vivo [45]. In order to test whether the our CRISPR–Cas12a system maintains precursor crRNA array processing and mediate efficient multiplex gene editing by only using a single customized CRISPR array in thermophilic fungus *M. thermophila*, we built a crRNA array expressing pre-crRNAs (array 1) in the order *cre1*–*res1*–*gh1*-*1* (Fig. 3b) as well as three single crRNA cassettes (Fig. 3a). We delivered the Cas12, crRNA (pooled crRNAs or array 1), and donor DNA in a one-step transformation (Fig. 3a, b). Then 20 or 22 transformants were picked randomly for PCR analysis (Additional file 3: Fig. S2). Some transformants showed two PCR bands (both of wild-type and knockout) were heterokaryon and subsequent separate of pure homokaryotic mutants from the transformants might be frequently necessary. Compared to the control transformation with donor DNAs alone (Additional file 4: Fig. S3), using both of Pooled single-crRNA-based and crRNA Array-based CRISPR–Cas12a systems, 40% (8 heterokaryons) and 32% (2 homokaryon + 6 heterokaryons) of the tested transformants displayed simultaneous triple homologous recombination efficiency in the three loci, *cre*-*1*, *res*-*1*, and *gh1*-*1*. In contrast to control experiment (0 out 23, 0% efficiency), pool- and array-based editing system showed the deletion efficiencies of *cre*-*1*, *res*-*1* and *gh1*-*1* were 35% (7 out 20) and 41% (9 out 22), 25% (5 out 20) and 32% (7 out 22), and 25% (5 out 20) and 27% (6 out 22), respectively (Table 1; Additional file 3: Fig. S2; Additional file 4: Fig. S3). This gene set has been tested using our CRISPR–Cas9 system (Additional file 5: Fig. S4), making it easy to compare the two systems (Table 1). By using our CRISPR–Cas9 system, 9 (including 3 homokaryons and 6 heterokaryons) out of 23 (39%) transformants displayed the triple-gene homologous recombination efficiency, in which 39%, 35% and 39% colonies showed deletions of *cre*-*1*, *res*-*1*, and *gh1*-*1*, respectively. The resulting triple-mutant Δ*cre1*Δ*res1*Δ*gh1*-*1* was named M3 and used as a host strain for subsequent engineering. Remarkably, all three target genes were equally well edited using either the crRNA array or pooled single-crRNA cassettes. Overall, these results demonstrate that the CRISPR–Cas12a system can efficiently mediate multiplex gene deletions in *M. thermophila* through a simple single crRNA array.

### CRISPR–Cas-assisted marker recycling (Camr) technology for iterative multiplex genome editing in consecutive steps

**Fig. 4:**
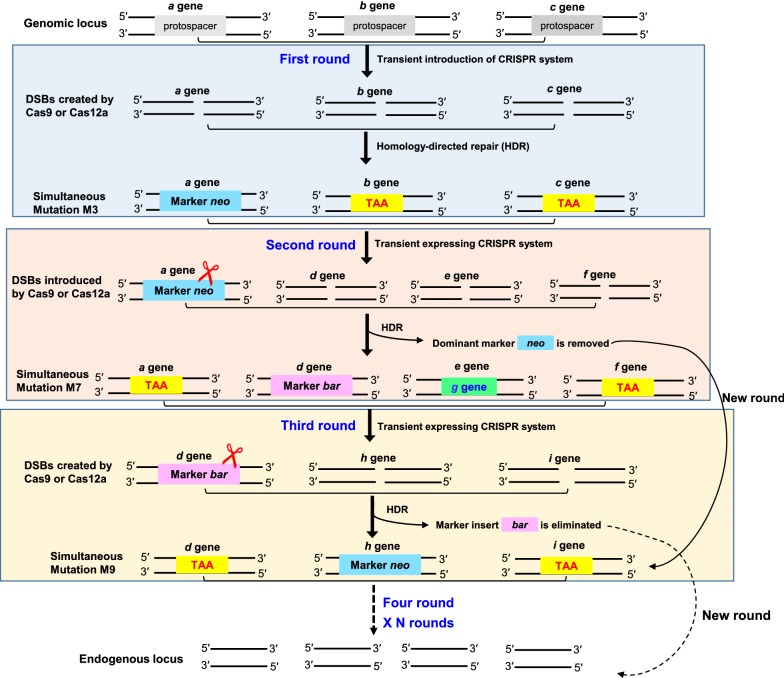
Schematic strategy of CRISPR–Cas-mediated marker recycling approach for iterative multiplex genome editing in *M. thermophila*. The hendecuple mutants were constructed in three successive transformations by alternatively using two selectable markers *neo* and *bar*. In the first transformation, a triple mutant was created by transient CRISPR–Cas12a/Cas9-mediated homology-directed repair (HDR). The *neo* cassette replaces an endogenous locus, and two target genes are both markerless deletions. In the second transformation, the insert of *neo* is removed and *bar* replaces a new endogenous locus in the triple-mutant host strain via HDR by the CRISPR–Cas12a/Cas9 system. In addition, two other gene loci undergo seamless gene replacement and markerless gene disruption. For the third transformation, the octuple mutant is selected for genetic manipulation using the CRISPR system. The selectable marker *neo* is inserted into a new endogenous locus, and the marker *bar* is scarlessly removed and the other new gene undergoes markerless deletion. This allows the marker *bar* to be used again in a new transformation round. DSBs, double-strand breaks; M, mutant

Cas nuclease-mediated double-strand breaks can efficiently improve the HR frequency when they occur in sites of interest and induce alterations. Hence, we reasoned that this recombination process could potentially rescue the selectable marker *neo*. The new crRNA target on *neo* could replace the existing *neo* cassette with any desired DNA sequence through CRISPR–Cas-assisted HDR. Thus, successive genome editing can be performed using only two selectable markers in a “ping-pong” style. We used this methodology to create a CRISPR–Cas-assisted marker recycling system (Fig. 4).

To test this idea, we selected nine key genes in the cellulase production pathway, including *cre*-*1* (MYCTH_2310085), *res*-*1* (MYCTH_2302052), *gh1*-*1* (MYCTH_115968), *alp*-*1* (MYCTH_2303011), *Mtxyr*-*1* (Mycth_2310145), *rca*-*1* (Mycth_2300719), *hcr*-*1* (Mycth_2309600), *ap*-*3* (Mycth_2307451), and *prk*-*6* (Mycth_2303559) [61‒65], and generated marker-recycling multiple knockouts or knockins using only two markers, *neo* and *bar*. For each round of transformations, either the single marker gene *neo* or *bar* was used for selection of antibiotic-resistant fungal transformation on medium plates supplemented with G418 (for *neo* gene resistance) or phosphinothricin (for *bar* gene resistance). In order to efficiently excise the marker gene in the next round transformation, we used only one marker and integrated this marker gene into a single random locus of target genes per each round of edits. In the first transformation, we targeted three genes including *cre*-*1*, *res*-*1*, and *gh1*-*1* and only locus of *gh1*-*1* was randomly selected for marker gene *neo* integration, which is convenient for *neo* knockout in successive transformation. In the second transformation, we targeted four genes (*neo*, *alp*-*1*, *rca*-*1* and *hcr*-*1*) and only locus of *alp*-*1* was selected for second marker gene *bar* integration, which is convenient for *bar* deletion in the third transformation. In successive transformation, we engineered three genes including *bar*, *ap*-*3* and *prk*-*6* and only locus of *ap*-*3* was selected for first marker gene *neo* integration, which is convenient for *neo* deletion in the next iteration.

**Fig. 5:**
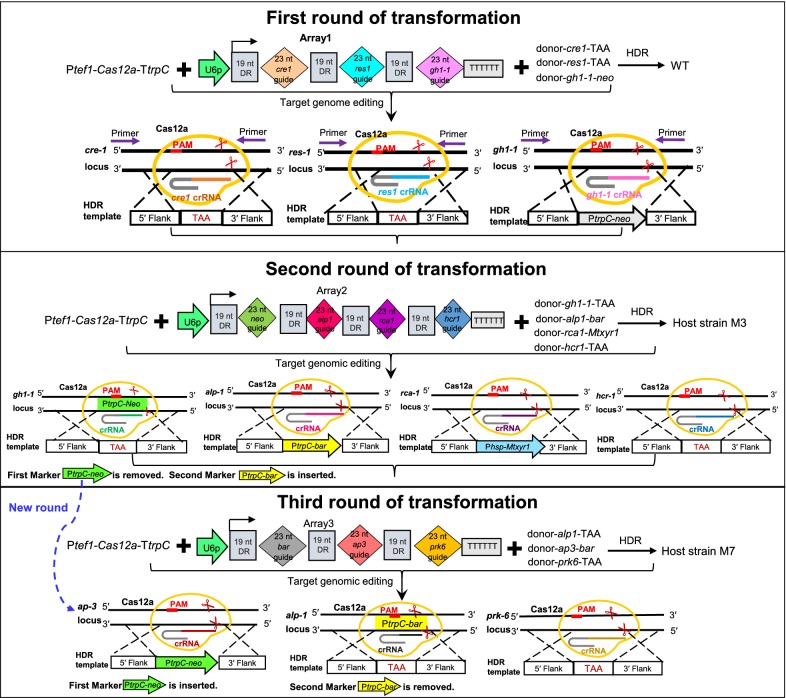
CRISPR–Cas12a-assisted marker recycling editing of eleven target genes, including nine genes involved in the cellulase production pathway, via three successive round transformations. The 11 genes were *cre*-*1*, *res*-*1*, *gh1*-*1*, *alp*-*1*, *xyr*-*1*, *rca*-*1*, *hcr*-*1*, *ap*-*3*, *prk*-*6*, and two selectable marker genes *neo* and *bar*. HDR, homology-directed repair; WT, wild type; DR, 19 nt direct repeat; U6p, U6 promoter; Ptef1, *tef1* promoter; TtrpC, *trpC* terminator. M, mutant strain. P*trpC*, *trpC* promoter. TAA, stop codon. TTTTTT, poly-T sequence

Three successive round of experiments were designed to test a proof-of-concept in generating nine genes mutants by using our “ping-pong” style of marker recycling strategy, in which the first maker gene *neo* can be eliminated in the second transformation after its initial use and then this *neo* gene can be used again in the third transformation, just as it was used in the first transformation. Based on this design, we can generate the nonuple mutant through three round of edits (Fig. 5). First, three genes, *cre*-*1*, *res*-*1*, and *gh1*-*1*, were targeted, resulting in the triple-mutant M3 (Figs. 3, 5). The second transformation was carried out with the host strain M3. We built a crRNA array expressing pre-crRNAs in the order *neo*–*alp1*–*rca1*–*hcr1* (array 2). After co-delivering Cas12a, array 2, and donor templates into M3, 23 putative transformants were picked randomly and analyzed for indels by PCR using primers flanking the targeted loci (Additional file 6: Fig. S5). We found that *alp*-*1* was replaced with the selectable marker *bar*, which conferred phosphinothricin resistance, whereas the marker *neo* and *hcr*-*1* were seamlessly deleted. In addition, the essential (hemi-)cellulase regulator *xyr*-*1* was inserted into the *rca*-*1* locus. Five colonies (1 homokaryon + 4 heterokaryon, 22%) were identified with these four loci modified, while none of colonies (0 out of 23, 0%) were shown simultaneous quadruple recombination in the control transformation with donor DNA alone (Table 1; Additional file 7: Fig. S6). In the second round transformation by using our CRISPR–Cas12a system, the gene disruption frequency of *neo*, *alp*-*1*, *rca1*-*1* and *hcr*-*1* were 26%, 30%, 13%, and 22%, respectively. We obtained four combinations of homokaryon mutation genotypes (Additional file 6: Fig. S5), namely one quadruple mutant Δ*cre1*Δ*res1*Δ*gh1*-*1*Δ*hcr1* (M4), one quintuple mutants Δ*cre1*Δ*res1*Δ*gh1*-*1*Δ*neo*Δ*alp1*Δ*hcr1* (M5 with disrupted *neo*) and one sextuple mutant Δ*cre1*Δ*res1*Δ*gh1*-*1*Δ*alp1*Δ*rca1*::*xyr1* (M6), and one septuple mutant Δ*cre1*Δ*res1*Δ*gh1*-*1*Δ*neo*Δ*alp1*Δ*rca1*::*xyr1*Δ*hcr1* (M7 with disrupted *neo*). The M5 and M7 mutants were very sensitive to G418, indicating that the marker *neo* was successfully removed and can be used again in the next genetic engineering operation.

Strain M7, which lacked the *neo* marker, was used for the third transformation. We constructed another crRNA array (array 3) to target three loci, the selectable marker *bar* and two other genes (*ap*-*3* and *prk*-6) involved in the cellulase production pathway. In the third round of HDR, the marker *bar* was excised, and *ap*-*3* was replaced by the marker *neo*, which was rescued in last round; *prk*-*6* was disrupted (Fig. 5). Nine transformants (1 homokaryon + 8 heterokaryons) showed HR in three gene loci and the efficiency of triple recombination was about 41% (Table 1; Additional file 8: Fig. S7), whereas none of transformants (0 out of 23) were displayed all triple-gene homologous recombination modified in control experiment with donor DNA alone (Table 1; Additional file 9: Fig. S8). We obtained three combinations of homokaryotic deletion strains, namely two octuple mutants Δ*cre1*Δ*res1*Δ*gh1*-*1*Δ*neo*Δ*alp1*Δ*rca1*::*xyr1*Δ*hcr1*Δ*bar*Δ*ap3* (M8-1, eight cellulase production-related genes edited with disrupted copies of *neo* and *bar*) and Δ*cre1*Δ*res1*Δ*gh1*-*1*Δ*neo*Δ*alp1*Δ*rca1*::*xyr1*Δ*hcr1*Δ*bar*Δ*prk6* (M8-2), and one nonuple mutant Δ*cre1*Δ*res1*Δ*gh1*-*1*Δ*neo*Δ*alp1*Δ*rca1*::*xyr1*Δ*hcr1*Δ*bar*Δ*ap3*Δ*prk*-*6* (M9, nine cellulase production pathway genes edited with disrupted copies of *neo* and *bar*; the functional *neo* was added into the *ap3* loci, whereas the selectable marker *bar* was rescued in this transformation round).

On the basis of these results for Cas12a, we tested and optimized our previously reported Cas9 system using the marker recycling approach to obtain more flexible Cas9-based mutagenesis in *M. thermophila* (Fig. 4). The genetic manipulation was performed by co-delivery of the PCR products of Cas9, sgRNAs, and donor DNA in three sequential rounds of transformation (Additional file 13: Fig. S5; Additional file 10: Fig. S9; Additional file 11: Fig. S10). The HR efficiency of each round in generating Cas9-mediated mutants is summarized in Table 1. The Cas9-based efficiency of simultaneously homologous recombination of three and four genes was approximately 38–39% and 22%, respectively, which were similarly observed in the transformation experiments by using array-based CRISPR–Cas12a system (Table 1), suggesting that crRNA array-based CRISPR–Cas12a system might be more cheaper and convenient for multiplex genome editing. Three successive rounds of transformations and selections resulted in one homokaryon nonuple mutant (M9, nine cellulase production pathway genes edited with disrupted copies of *neo* and *bar*), in which gain-of-function of *neo* but loss-of function of *bar* were confirmed.

Together, these results indicate that the CRISPR–Cas12a and CRISPR–Cas9 systems are both efficient tools for mediating marker recycling in *M. thermophila*. We named this CRISPR–Cas12a/Cas9-assisted marker recycling system as “Camr” technology.

### Evaluation of cellulolytic enzyme production in *M. thermophila* mutants obtained via our Camr technology

**Fig. 6:**
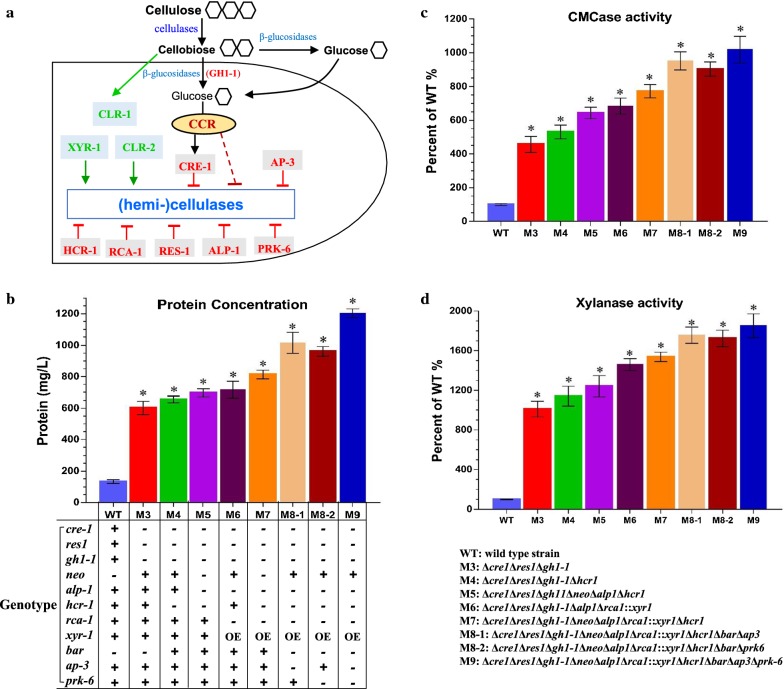
Cellulolytic phenotype analysis of eight deletion mutants and wild-type strain (WT). **a** Schematic model illustrating the coordinated action of cellulolytic factors under cellulose induction. Under cellulose conditions, the positive regulators CLR-1, CLR-2, and XYR-1 have been identified as key transcription factors regulating cellulose degradation. Preferred carbon sources such as glucose activate the essential transcription factor CRE-1, which represses the expression of cellulase genes, a phenomenon called carbon catabolite repression (CCR). Lack of the major intracellular β-glucosidase GH1-1 facilitates the accumulation of intracellular cellobiose, which can trigger signaling cascades that include expression of cellulase genes repressed by CRE-1 and activated by CLR-1/2 and XYR-1. The newly identified regulators RES-1, HCR-1, RCA-1, AP-3, the major secreted protease ALP-1, and protein kinase PRK-6 were shown to negatively affect cellulase production. **b** Secreted protein production of all strains after 6-days cultivation in 2% Avicel medium supplemented with 0.75% yeast extract. Genotype of the eight deletion mutants as compared to the WT strain. The 11 target genes edited by our CRISPR–Cas12a system included *cre*-*1*, *res*-*1*, *gh1*-*1*, *neo*, *alp*-*1*, *rca*-*1*, *xyr*-*1*, *hcr*-*1*, *bar*, *ap*-*3* and *prk*-*6*. The symbol of +, or − or OE indicated the target gene is present, or deleted or overexpressed. **c**, **d** Assays for CMCase and xylanase activity in the eight deletion mutants and WT strains in 2% Avicel plus 0.75% yeast extract after 6 days culture. Bars marked by asterisks in each group differ significantly from the unmarked bars (Tukey’s HSD, *p *< 0.001). Error bars represent SD from three replicates

The quadruple mutant Δ*cre1*Δ*res1*Δ*gh1*-*1*Δ*alp1* from our previous study exhibited a pronounced hyper-cellulase secretion phenotype [20], therefore, we used it to assess whether the dose effects of other cellulolytic factors such as *xyr*-*1*, *rca*-*1*, *hcr*-*1*, *ap*-*3* and *prk*-*6* (Fig. 6a) would further enhance cellulase production in this quadruple mutant. We examined the cellulolytic phenotypes on cellulose medium of the eight mutants obtained through the three sequential rounds of transformation described above. As expected, all eight mutants displayed significantly enhanced secreted protein production (~ 602.2 to 1201.9 mg L^−1^) compared with the wild type WT strain (~ 133.1 mg L^−1^); in particularly, the secretome of M9 (~ 1201.9 mg L^−1^) was approximately 9.0-fold higher than that of the WT (Fig. 6b; Additional file 12: Fig. S11). Consistent with the increased secreted protein levels, cellulolytic enzyme activity in the eight mutants was remarkably higher than in WT (Fig. 6c, d). Notably, the endoglucanase and xylanase activity in mutants M8-1, M8-2, and M9 was 9.5- to 10.1-fold and 17.2- to 18.5-fold higher, respectively, than in the WT. Together, these results indicate that the dose-controlled pathway of cellulase expression and secretion is a promising strategy for cellulolytic fungi to develop enzyme hyper-producers via our Camr technology.

## Discussion

A first-generation genome editing tool using the CRISPR–Cas9 system was developed in thermophilic fungi *M. thermophila* and *M. heterothallica* [20]. This CRISPR–Cas9 system was difficult to use when there were more than five target genes because all the sgRNA-expressing cassettes had to be constructed and delivered at the same time. This issue was addressed by the CRISPR–Cas12a system. Although the CRISPR–Cas12a system has been intensively and successfully utilized in higher eukaryotes for genome editing [44–49], this system has been comparatively underexplored in microbes. Until now, Cas12a-based genomic editing tools have been demonstrated only in yeast, a few fungi species [50, 51, 57, 58], and several bacteria [52–55], and have not yet been widely established in thermophilic filamentous fungi. While this manuscript was in revision, a functional CRISPR-Cpf1 system for gene editing was published in *T. thermophilus* (synonym of *M. thermophila*) [58] demonstrating that this system may work widely in filamentous fungi.

In this study, we developed a Cas12a-based system from *Acidaminococcus* sp. BV3L6 as an efficient genomic editing tool for the thermophilic fungus *M. thermophila* (Fig. 1). We found that the CRISPR components of the Cas12a and crRNA expression cassettes were able to function when introduced transiently and without direct selection. PCR analysis indicated that the CRISPR–Cas12a cassettes were not integrated into the genome (data not shown). This transiently expressed CRISPR–Cas12a system efficiently enabled the generation of indel mutations in the *M. thermophila* genome (Figs. 2, 5). The transient CRISPR–Cas9 system produced similar results in *M. thermophila* (Additional file 13: Fig. S5; Additional file 10: Fig. S9; Additional file 11: Fig. S10). These data are consistent with previous reports in *Magnaporthe oryzae* and *Candida albicans*, which showed the transient introduction of the CRISPR–Cas9 system efficiently mediated gene editing [24, 60]. The efficient and successful application of transient CRISPR–Cas technology has useful implications. First, this system may minimize the problems associated with Cas toxicity (such as Cas9 and FnCpf1) in some fungi species [24, 50] and off-target cleavage activity. Second, gene edited mutants can be generated rapidly using PCR-amplified cassettes, which obviates the need to construct Cas12a or Cas9 expressing strains, thereby dramatically reducing the time, cost, and use of selectable marker. Although multiplex editing of homologous recombination genes occurred a little more frequently in colonies transformed with pooled crRNAs than in those transformed with array1, the Cas12a-mediated system produced frequencies similar to those of the Cas9 system in colonies transformed with array 1 (Table 1). In addition, the three target genes, *cre*-*1*, *res*-*1*, and *gh1*-*1*, were equally edited and the editing efficiency of each gene was not affected by using either a single-array approach or pooled cassettes. Previous reports in mammalian and yeast cells have found that the position of the crRNA on the array and the number of target genes (up to four) were not crucial for editing efficiency [45, 50]. Thus, we used a single-array approach to perform the Cas12a-mediated multiplex gene editing in the further manipulations. Our results expand the genome editing toolbox to efficiently generate mutations in filamentous fungi.

Previously, we obtained multiple deletion mutants with G418-resistance marker *neo*, which was integrated simultaneously into each target loci via the CRISPR–Cas9 system, and the copy number of the integrated marker was in good accordance with the number of target genes [20]. Hence, we reasoned that triple or quadruple mutants could be generated by integrating a selectable marker in only one target locus via the CRISPR–Cas12a/Cas9 system, thereby minimizing the copy number of the marker gene, and creating seamless gene deletion or insertion in other target loci. Our results confirm that the acquisition of triple-mutant M3 was achieved by integrating the selectable marker gene *neo* at the *gh1*-*1* locus and that markerless deletion of *cre*-*1* and *res*-*1* was achieved via one-step transformation using either the CRISPR–Cas12a or CRISPR–Cas9 systems. Additionally, our data indicate that the recombination efficiencies of the target genes did not rely on the integration of a selective marker into the locus, allowing for efficient markerless genomic modification as desired. A similar strategy could be used to exchange or modify target promoters to alter gene expression for industrial applications.

New approaches for marker recycling based on the powerful CRISPR–Cas9 system have been demonstrated recently in fungi, namely *S. cerevisiae*, *C. albicans*, *A. oryzae* and *A. niger* [36, 37, 61, 62]. Most of these approaches combined Cas9 editing with the auxotrophic marker *pryG*/*ura3* or recombinase-promoted excision. These methods require additional steps for either constructing auxotrophic strains or autonomous replicating plasmids carrying the corresponding recombinase [36, 37, 62], which is complicated and time-consuming. Our results for generating the nonuple mutant M9 (Fig. 5) by marker recycling and iterative stacking of traits in the same strain through editing both the previously used marker and other different loci via the CRISPR/Cas system is a feasible new approach. We employed this strategy to design our Camr technology through alternative use of two selectable markers in a “ping-pong” style where the first marker is rescued when the second marker is used (Fig. 4). As shown in Table 1, the Cas9-based efficiency of simultaneously homologous recombination of three and four genes was approximately 38%, 39% and 22%, respectively, which were similarly observed in the transformation experiments by using array-based CRISPR–Cas12a system, suggesting that crRNA array-based CRISPR–Cas12a system might be more cheap and convenient for multiplex genome editing. Recent work by Zhang and co-workers performed a computational analysis of the targeting range of the CRISPR–Cas12a system and CRISPR–Cas9 system in the human genome [63]. The authors demonstrated that the targeting range of wild-type Cas12a to one target site was per ~ 33 bp in human coding sequences, while the targeting range of Cas9 to one cleavage site was per ~ 7 bp in human coding sequences, suggesting the target frequency of Cas12a is more rare than Cas9 [63]. However, Zhang and co-workers engineered Cas12a variants to exist with altered PAM specificities including 5′ TYCV and 5′ TATV, 5′ MCCC and 5′ RATR, which have expanded the targeting range of Cas12a to one target site per ~ 7 bp in human coding sequences [63]. Moreover, the T-rich PAMs of the Cas12a allow for applications in genome editing in organisms with particularly AT-rich genomes or sequence areas of interest with AT enrichment, so the T-rich-dependent PAMs of Cas12a proteins expand the targeting range of genome editing nucleases and become a useful complement to CRISPR–Cas9 system for genetic engineering.

The traditional methods in filamentous fungi for obtaining multiple gene mutants from continuous single deletion engineering (usually per ~ 4‒5 weeks) to multiple genes are very complicated, laborious and time-consuming, and also limited in the ability to knock out multiple genes at the same time. Compared with the utilization of conventional methods to edit multiple gene, our Camr technology can significantly reduce the time and labor to engineer multiple genes (more than 10) via two or three successive transformations. The manipulation time for our procedure was reduced to 6 days for each round of modification, and up to three or four gene targets can be modified simultaneously. For instance, the triple-gene disruptions M3 mutant (Δ*cre1*Δ*res1*Δ*gh1*-*1*) was obtained by our Camr technology within 6 days and an additional 7 days were required for fungal conidia growth. We can also obtain multiple mutant genotypes with different combinations of disrupted four genes, such as M4, M5, M6, and M7, in second round transformation (time spending another 6 + 7 days from the host strain M3), which provides additional opportunities to obtain desired mutant strains of industrial interest and thus greatly improves the efficiency and time of genome editing. Taken together, we successfully targeted nine genes in the cellulase production pathway and generated the mutant M9 within ~ 5 weeks from the wild-type strain, in which all nine selected genes were edited correctly, via three successive transformations using markers *neo* and *bar* in our Cas12a and Cas9 systems (Fig. 5).

In saprophytic fungi, lignocellulolytic enzyme production is mainly regulated at both the transcriptional and post-translational levels which involves combinatorial action of several transcriptional activators and repressors [1, 2, 64–67]. Genetically engineering the regulatory network at both levels, such as overexpression of transcriptional activators and deletion of repressors, represents an efficient and promising strategy for significantly improving cellulases production in cellulolytic fungi including *T. reesei*, *Penicillium oxalicum*, *N. crassa* and *M. thermophila* [1, 7, 20, 68]. Generally, the cellulase production is induced by cellulose-derived oligosaccharides (e.g., cellobiose) and repressed by preferentially utilized saccharides (e.g., glucose), a phenomenon called carbon catabolite repression (CCR). Therefore, the carbon catabolite repressor CreA/Cre1 is a well-known highly conserved cellulase repressor throughout the fungal kingdom and elimination of the function of CreA/Cre1 resulted in significantly improved lignocellulolytic enzyme production in *Aspergillus* spp., *T. reesei*, *N. crassa*, *P. oxalicum* and *M. thermophila* [1, 2, 7, 20, 68]. Additionally, elimination of β-glucosidases which hydrolyze cellobiose to glucose is able to improve the expression and production of cellulolytic enzymes. For example, disruption of the major intracellular β-glucosidase enhanced cellulase production on cellulose in *N. crassa* [1], *P. oxalicum* [7, 69] and *M. thermophila* [20]. In addition, our recent studies in *N. crassa* demonstrated that single deletion of the transcriptional regulator *res*-*1* [66], *hcr*-*1* [70] or *rca*-*1* [64], adaptor protein *ap*-*3* [67] or protein kinase *prk*-*6* [67] improved cellulase production and activities under cellulolytic condition.

Our previous report in *M. thermophila* [20] showed that the single deletion of *cre*-*1*, *gh1*-*1*, *res*-*1*, or *alp*-*1* resulted in significant improvement of cellulase production by about 3.3-, 2.3-, 2.6- or 2.1- fold higher amounts (~ 381.8, ~ 266.2, ~ 306.6 or ~ 245.2 mg L^−1^) compared with WT strain under 5 days 2% Avicel cultivation, implying that CRE-1 play the largest phenotypic effect on cellulase production in *M. thermophila*. Based on these above obvious improvement, we hypothesized that there might be a significant synergistic and additive reinforcement effect in cellulase production by genetic integrating modification of these cellulolytic factors. Therefore, in this study, we generated the mutants M3 (Δ*cre1*Δ*res1*Δ*gh1*-*1*), M7 (M3 + Δ*neo*Δ*alp1*Δ*rca1*::*xyr1*Δ*hcr1*), and M9 (M7 + Δ*bar*Δ*ap3*Δ*prk*-*6*) through using our Camr technology. As expected, the cellulase secretion and activity phenotypes of M3 (~ 602.2 mg L^−1^, ~ 4.5-fold), M7 (~ 813.5 mg L^−1^, ~ 6.1-fold) and M9 (~ 1201.9 mg L^−1^, ~ 9.0-fold) were significantly enhanced compared with those of the WT strain (~ 133.1 mg L^−1^) (Fig. 6), suggesting that the key regulator CRE-1 play a master role in improving cellulase production and other factors also have positive and synergistic effects on increase of cellulase production. As depicted above, since one key regulator CRE-1 has greatly profound effects on cellulase expression and secretion in *M. thermophila* and the resulting strain M3 led to significantly higher cellulase production. Thus, the latter engineered strains M7 and M9 produced moderately enhanced cellulase production due to this very high background level of host strain M3.

These enhanced cellulase production levels by the engineered strains developed here are comparable to those of strains engineered in the other well-known cellulase production species *P. oxalicum* [69], in which the quadruple mutants RE-29 (deleting *bgl2* and *creA*, along with over-expressing the gene *clrB* and *xlnR*/*xyr1*) showed more cellulolytic enzyme activities and secretion abilities than the triple-mutant RE-10 (deleting *bgl2* and *creA*, along with over-expressing the gene *clrB*). This phenomenon suggests that engineering the key factors involved cellulolytic regulatory networks perform the synergistic improvement of cellulase production. In summary, this result indicates that the regulatory network of cellulase expression and secretion can be genetically engineered using the Camr technology as a simple and efficient strategy to improve cellulase production in cellulolytic fungi within a short time. Therefore, the Camr approach can be used for iterative genomic manipulations in both basic research and industrial strain engineering for biotechnological applications.

## Conclusions

In this study, we report an *Acidaminococcus* sp. Cas12a-based CRISPR system for multiplex genome editing, using a single-array approach in thermophilic filamentous *M. thermophila*. These CRISPR–Cas12a cassettes worked well for simultaneous multiple gene deletions/insertions. We also developed new approaches that allow easy and efficient marker recycling and iterative stacking of traits in the same thermophilic fungus strain either, using the newly established transiently expressed CRISPR–Cas12a system or the established CRISPR–Cas9 system to make DNA breaks in selected markers. Together we called them CRISPR–Cas-assisted marker recycling technology (Camr technology). We demonstrated its performance by targeting nine genes involved in the cellulase production pathway in *M. thermophila* via three transformation rounds, using two selectable markers *neo* and *bar*. The nonuple mutant M9 were then obtained, in which protein productivity and lignocellulase activity were 9.0- and 18.5-fold higher than in the wild type. We expect this advance to accelerate biotechnology-oriented engineering processes in fungi.

## Methods

### Strains and growth conditions

*Myceliophthora thermophila* ATCC 42464 was obtained from the American Type Culture Collection (ATCC). *M. thermophila* strains were cultured on Vogel’s MM supplemented with 2% sucrose at 45 °C for 7 days to obtain conidia. Antibiotics were added when needed to screen for transformants. For flask culture, *M. thermophila* conidia at 10^6^ mL^−1^ were inoculated in 100 mL medium (containing 1 × Vogel’s salt, 2% Avicel, and 0.75% yeast extract) at 45 °C with shaking at 150 rpm. For vector manipulation and propagation, *Escherichia coli* DH5α (Invitrogen, Shanghai, China) was cultured at 37 °C in Luria–Bertani broth with kanamycin or ampicillin (100 μg mL^−1^) for plasmid selection.

### Plasmid construction for genetic engineering

All the primer sequences used in this study are listed in Additional file 1: Table S1. All the PCR products were amplified using Phusion high-fidelity DNA polymerase (Thermo Fisher, Waltham, MA, USA).

To generate the Cas12a expression plasmid, a codon-optimized *Cas12a* gene (*AsCpf1*, GenBank: U2UMQ6) with attached HAC-1 (GenPept: MYCTH_2310995) and SV40 nuclear localization signal (NLS-*Cas12a*-NLS) was synthesized artificially by the Life Science Research Services Company (Genewiz, Suzhou, China). The strong constitutive *tef1* (translation elongation factor EF-1, MYCTH_2298136) promoter P*tef1* of *M. thermophila* was used to express *Cas12a*. The T*trpC* terminator was cloned from the vector pNA52-1N (GenBank number: Z32697). The synthetic NLS-*Cas12a*-NLS, P*tef1* promoter [20], and *TtrpC* terminator were amplified and assembled into a p0380-bar plasmid [41] to form a P*tef1*-*Cas12a*-*TtrpC* cassette by using a NEB Gibson assembly kit. The sequence of P*tef1*-*Cas12a*-*TtrpC* expressing cassette is provided in Additional file 13.

The crRNA expression cassette comprised the *M. thermophila* U6 promoter [20], the target sequence of 23 nt, a short direct repeat of 19 nt (5′-AATTTCTACTCTTGTAGAT-3′) [43] and ploy T sequence used as terminator. The target genes were *amdS* (GenBank: M16371.1), *cre*-*1* (MYCTH_2310085), *res*-*1* (MYCTH_2302052), *gh1*-*1* (MYCTH_115968), *alp*-*1* (MYCTH_2303011), *rca*-*1* (Mycth_2300719), *hcr*-*1* (Mycth_2309600), *ap*-*3* (Mycth_2307451), *prk*-*6* (Mycth_2303559), and the selectable markers *neo* (GenBank: HQ416708) and *bar* (GenBank: X17220). Specific crRNA target sites were designed by using the CRISPR-offinder tool [71], which is available at the BiooTools website (http://www.biootools.com). The target sequence with low off-target probability were selected. The 23-nt protospacer sequences of guide crRNAs are provided in Additional file 14: Table S2. Briefly, the single crRNA expression cassettes, U6p-*amdS*-crRNA, U6p-*cre1*-crRNA, U6p-*res1*-crRNA, and U6p-*gh1*-*1*-crRNA, were generated by PCR amplification with primer pairs in Additional file 1: Table S1 and cloned into vector pJET1.2 for sequencing (CloneJET PCR Cloning Kit; Thermo Fisher Scientific, Waltham, MA USA). Three crRNA arrays expressed by a U6 promoter (U6p-array1-*cre1*-*res1*-*gh1*-*1*, U6p-array2-*neo*-*alp1*-*rca1*-*hcr1* and U6p-array3-*bar*-*ap3*-*prk6*) were synthesized by the Genewiz gene synthesis service. Sequence for the all the single crRNAs and crRNA arrays are provided in Additional file 15.

To construct plasmids expressing sgRNA, specific sgRNA target sites in *cre*-*1*, *res*-*1*, *gh1*-*1*, *alp*-*1*, *rca*-*1*, *hcr*-*1*, *ap*-*3*, *prk*-*6* were identified using the sgRNACas9 tool [72] with the *M. thermophila* genome sequence and the target gene as the inputs. The target sequence with low off-target probability were selected. The sgRNA expression cassettes, U6p-*rca1*-sgRNA, U6p-*hcr1*-sgRNA, U6p-*ap3*-sgRNA, U6p-*prk6*-sgRNA, U6p-*neo*-sgRNA, and U6p-*bar*-sgRNA, were constructed as described previously [20]. The target sequences for all the sgRNAs are provided in Additional file 2: Table S2.

To construct the donor DNAs, the 5′ and 3′ flanking fragments of *cre*-*1* (600 bp/539 bp), *res*-*1* (600 bp/600 bp), *gh1*-*1* (600 bp/600 bp), *alp*-*1* (600 bp/600 bp), *rca*-*1* (522 bp/608 bp), *hcr*-*1* (600 bp/600 bp), *neo* (563 bp/600 bp), *ap*-*3* (600 bp/580 bp), *prk*-*6* (547 bp/600 bp), and *bar* (599 bp/560 bp) were amplified separately. The selectable marker cassettes P*trpC*-*neo* and P*trpC*-*bar* were amplified from the p0380-neo and p0380-bar plasmids [41]. To construct the markerless templates, the 5′ and 3′ fragments were assembled and ligated into pUC118 using a NEB Gibson kit to generate donor-*cre1*-TAA, donor-*res1*-TAA, donor-*neo*-TAA, donor-*hcr1*-TAA, donor-*prk6*-TAA, and donor-*bar*-TAA. The “TAA” in the donor DNA indicated introducing a stop codon in the target genes. The 5′ and 3′ fragments and P*trpC*-*neo* or P*trpC*-*bar* were assembled and inserted into pUC118 to generate donor-*cre1*-neo, donor-*gh1*-*1*-*neo*, donor-*alp1*-*bar*, and donor-*ap3*-*neo*. To overexpress *Mtxyr*-*1* (Mycth_2310145) in the *rca*-*1* locus, the 1200-bp promoter of *hsp70* (heat shock protein 70, Mytcth_112686) and the full-length sequence of *Mtxyr*-*1* were amplified separately. These two fragments and the 5′ and 3′ fragments of *rca*-*1* were assembled and ligated into the pUC118 using Gibson kit to generate donor-*rca1*-*Mtxyr*-*1*.

### Transformation of *M. thermophila* protoplasts

The PEG-mediated transformation of *M. thermophila* protoplasts was performed as described previously [41]. For *amdS* mutagenesis, the *amdS* expression strain M1 [20] was used as the host strain. Briefly, 10 μg of the PCR products of P*tef1*-*Cas12a*-*TtrpC* (9.2 μg) and U6p-*amdS*-crRNA (0.8 μg) at the same molar concentration ratio were co-transformed into M1 protoplasts. The *amdS* mutants were inoculated onto MM plates that included 2 mg mL^−1^ fluoroacetamide (FAA). FAA-resistant mutants were isolated and tested for growth on acetamide medium, followed by PCR sequencing. For *cre*-*1* deletion, total 10 μg PCR cassettes of P*tef1*-*Cas12a*-*TtrpC* (6.8 μg), U6p-*cre1*-crRNA (0.6 μg), and donor-*cre1*-*neo* (2.6 μg) were mixed at the same molar concentration ratio and added to the protoplasts of WT. Transformants were screened for *neo* resistance with 80 μg mL^−1^ G418 after 3 days of culture, followed by PCR identification.

For multiplex gene editing by Cas12a using pooled single or array crRNAs, the 11.5‒12.4 μg PCR products of three pooled crRNAs cassettes (0.5 μg for each crRNA) or array 1 (0.6 μg) and donor DNAs of *cre*-*1* (1.2 μg), *res*-*1* (1.2 μg), and *gh1*-*1* (2.5 μg) were mixed with P*tef1*-*Cas12a*-*TtrpC* (6.0 μg) at the same molar concentration ratio and co-transformed into WT protoplasts. For multiple gene editing with the transient CRISPR–Cas9 system, the 12.7 μg PCR products of sgRNAs (0.6 μg for each sgRNA), donor DNAs of *cre*-*1* (1.2 μg), *res*-*1* (1.2 μg), and *gh1*-*1* (2.5 μg), and P*tef1*-*Cas9*-*TtrpC* (6.0 μg) were co-transformed into WT protoplasts. The putative transformants were selected with 80 μg mL^−1^ G418 and confirmed by PCR, generating the triple deletion strain Δ*cre1*Δ*res1*Δ*gh1*-*1* (M3).

To remove marker cassette *neo*, second round manipulation was performed with the transient Cas12a or Cas9 system. Briefly, total ~ 17.0‒19.0 μg PCR cassettes of array 2 (0.65 μg) or sgRNAs (0.6 μg for each sgRNA) and P*tef1*-*Cas12a*-*TtrpC* (6.0 μg) or P*tef1*-*Cas9*-*TtrpC* (6.0 μg) were mixed with donor DNA cassettes of *neo* (1.2 μg), *alp*-*1* (2.5 μg), *rca*-*1* (5.5 μg), and *hcr*-*1* (1.2 μg) and co-transformed into protoplasts of the M3 strain. Putative transformants were selected on phosphinothricin (100 μg mL^−1^) and confirmed by PCR, creating the septuple deletion strain Δ*cre1*Δ*res1*Δ*gh1*-*1*Δ*neo*Δ*alp1*Δ*rca1*::*xyr1*Δ*hcr1* (M7). For third round manipulation, the 11.5‒12.7 μg PCR products of array 3 (0.6 μg) or sgRNAs (0.6 μg for each sgRNA) and P*tef1*-*Cas12a*-*TtrpC* (6.0 μg) or P*tef1*-*Cas9*-*TtrpC* (6.0 μg) were mixed with donor DNAs of *bar* (1.2 μg), *ap*-*3* (2.5 μg) and *prk*-*6* (1.2 μg) and co-transformed into M7 protoplasts. Putative transformants were selected on G418 for 3 days and confirmed by PCR.

For single gene editing, control experiments were performed by adding 2.6 μg of donor-cre1-*neo* alone, or only the Cas12a cassette (6.8 μg) and donor-cre1-*neo* (2.6 μg), or only U6p-cre1-crRNA (0.6 μg) and donor-cre1-*neo* (2.6 μg) to the fungal protoplasts. For multiplex gene editing, control experiments were performed by adding 4.9‒10.4 μg of donor DNAs alone without CRISPR–Cas9/12a expression cassettes to the fungal protoplasts. Transformants were screened for *bar* resistance with phosphinothricin (100 μg mL^−1^) or *neo* resistance with G418 (80 μg mL^−1^), followed by PCR identification with paired primers (Additional file 1: Table S1).

### Protein and enzyme assays

The protein concentration in the supernatants was determined using a Bio-Rad protein assay kit (Bio-Rad, Hercules, CA, USA). Absorbance was measured at 595 nm and bovine serum albumin was used as the standard. For protein gel electrophoresis, 20-μL unconcentrated culture supernatant was loaded onto a polyacrylamide gel (Novex^®^ NuPAGE^®^ Pre-cast Protein Gels, Thermo Fisher Scientific) for sodium dodecylsulfate-polyacrylamide gel electrophoresis (SDS-PAGE). Endoglucanase and endo-1,4-β-xylanase activity in the culture supernatants was determined using an Azo-cm-cellulose assay kit (Megazyme) and an Azo-xylan kit (Megazyme) in accordance with the manufacturer’s instructions. All estimates were performed in three repeated assays. The statistical significance of differences among WT and mutant strains was assessed by one-way analysis of variance.

## Supplementary information

**Additional file 1: Table S1.** List of PCR primers used in this study.

**Additional file 2: Figure S1.** Verification of *cre*-*1* gene deletions in selected transformants with co-transformation of only Cas12a and donor DNA, only crRNA and donor DNA, or only donor DNA. (A) Schematic of homologous recombination (HR) of target gene cre-1. (B–D) PCR analysis of *cre*-*1* deletion with one primer (cre1-out-F) located upstream of the 5′ flanking region of genomic DNA and the other (cre1-in-R) located in the 3′ flanking region of genomic DNA. The expected length of disrupted transformants was 1.9 kb, while that of the WT host strain, used as a negative control, was 1.0 kb (rightmost lane). Heterokaryotic transformants showed two PCR bands (both of wild-type and knockout). HDR, homology-directed repair; WT, wild type.

**Additional file 3: Figure S2.** Verification of triple-gene deletions of *cre*-*1*, res-1 and *gh1*-*1* in selected transformants by using Pooled single-crRNA-based CRISPR–Cas12a system (A) or crRNA Array-based CRISPR–Cas12a system (B). PCR analysis of triple-gene deletion of *cre*-*1*, res-1 and *gh1*-*1* in selected transformants using one primer (cre1/res1/gh1-1-out-F) located upstream of the 5′ flanking region of genomic DNA and the other primer (cre1/res1/gh1-1-in-R) located in the 3′ flanking region of genomic DNA. The expected lengths of disrupted transformants of *cre*-*1*, *res*-*1* and *gh1*-*1* were 0.8, 0.7 and 1.9 kb, respectively, while those of WT strain (rightmost lane) was 1.2, 0.9 and 1.0 kb, respectively. Heterokaryotic transformants showed two PCR bands (both of wild-type and knockout). The symbol of star indicated deletion mutant. HDR, homology-directed repair; WT, wild type. U6p, U6 promoter; Ptef1, *tef1* promoter; TtrpC, *trpC* Terminator.

**Additional file 4: Figure S3.** Verification of triple-gene deletions of *gh1*-*1*, *cre*-*1* and *res*-*1* in selected transformants with co-transformation of only three donor-DNAs without CRISPR expressing cassettes. (A) Schematic of homologous recombination (HR) of target genes mediated by each donor DNA. (B) PCR analysis of triple-gene deletion of *gh1*-*1*, *cre*-*1* and *res*-*1* in selected 23 transformants using one primer (cre1/gh1-1/res1-out-F) located upstream of the 5′ flanking region of genomic DNA and the other primer (cre1/gh1-1/res1-in-R) located in the 3′ flanking region of genomic DNA. The expected lengths of disrupted transformants of *gh1*-*1*, *cre*-*1* and *res*-*1* were 1.9, 0.8 and 0.7 kb, respectively, while those of the host strain (rightmost lane) was 1.0, 1.2 and 0.9 kb, respectively. Heterokaryotic transformants showed two PCR bands (both of wild-type and knockout).

**Additional file 5: Figure S4.** First round of target genomic editing by CRISPR–Cas9 system. (A) Schematic of homologous recombination (HR) of *cre*-*1, res*-*1* and *gh1*-*1* mediated by Cas9, sgRNAs and donor DNA. (B) PCR analysis of triple-gene deletion of *cre*-*1*, *res*-*1* and *gh1*-*1* in selected transformants using one primer (cre1/res1/gh1-1-out-F) located upstream of the 5′ flanking region of genomic DNA and the other primer (cre1/res1/gh1-1-in-R) located in the 3′ flanking region of genomic DNA. The expected lengths of disrupted transformants of cre-1, res-1 and gh1-1 were 0.8, 0.7 and 1.9 kb, respectively, while those of WT strain (rightmost lane) was 1.2, 0.9 and 1.0 kb, respectively. Heterokaryotic transformants showed two PCR bands (both of wild-type and knockout). The symbol of star indicated deletion mutant. HDR, homology-directed repair; WT, wild type.

**Additional file 6: Figure S5.** Second round of target genomic editing by CRISPR–Cas12a system. (A) Schematic of homologous recombination (HR) of *neo*, *alp*-*1*, *rca*-*1* and *hcr*-*1* mediated by Cas12a, array2 and donor DNA. (B) PCR analysis of quadruple-gene deletion of *neo, alp*-*1, rca*-*1* and *hcr*-*1* in selected transformants using one primer (gh1-1-out-F2, alp1/rca1/hcr1-out-F) located upstream of the 5′ flanking region of genomic DNA and the other primer (gh1-1-in-R2, alp1/rca1/hcr1-in-R) located in the 3′ flanking region of genomic DNA. The expected lengths of disrupted transformants of *neo, alp1, rca*-*1* and *hcr*-*1* were 0.8, 1.6, 5.0 and 0.7 kb, respectively, while those of the host strain (rightmost lane) was 1.9, 1.0, 0.6 and 1.0 kb, respectively. Heterokaryotic transformants showed two PCR bands (both of wild-type and knockout). The symbol of star indicated deletion mutant. HDR, homology-directed repair; symbol star indicated deletion mutant.

**Additional file 7: Figure S6.** Verification of quadruple-gene deletions of *neo*, *alp*-*1*, *rca*-*1* and *hcr*-*1* in selected transformants with co-transformation of only four donor-DNAs without CRISPR expressing cassettes. (A) Schematic of homologous recombination (HR) of target genes mediated by each donor DNA. (B) PCR analysis of quadruple-gene deletion of *neo*, *alp*-*1*, *rca*-*1* and *hcr*-*1* in selected transformants using one primer (gh1-1-out-F2, alp1/rca1/hcr1-out-F) located upstream of the 5′ flanking region of genomic DNA and the other primer (gh1-1-in-R2, alp1/rca1/hcr1-in-R) located in the 3′ flanking region of genomic DNA. The expected lengths of disrupted transformants of *neo*, *alp1*, *rca*-*1* and *hcr*-*1* were 0.8, 1.6, 5.0 and 0.7 kb, respectively, while those of the host strain (rightmost lane) was 1.9, 1.0, 0.6 and 1.0 kb, respectively. Heterokaryotic transformants showed two PCR bands (both of wild-type and knockout).

**Additional file 8: Figure S7.** Third round of target genomic editing by CRISPR–Cas12a system. (A) Schematic of homologous recombination (HR) of *bar*, *ap*-*3* and *prk*-*6* mediated by Cas12a, array2 and donor DNA. (B) PCR analysis of triple-gene deletion of bar, ap3 and prk6 in selected 22 transformants using one primer (alp1-out-F2, ap3/prk6-out-F) located upstream of the 5′ flanking region of genomic DNA and the other primer (alp1-in-R2, gh1-1/res1-in-R) located in the 3′ flanking region of genomic DNA. The expected lengths of disrupted transformants of *bar*, *ap*-*3* and *prk*-*6* were 0.8, 2.0 and 0.8 kb, respectively, while those of the host strain (rightmost lane) was 2.0, 1.2 and 1.2 kb, respectively. Heterokaryotic transformants showed two PCR bands (both of wild-type and knockout). The symbol of star indicated deletion mutant. HDR, homology-directed repair.

**Additional file 9: Figure S8.** Verification of triple-gene deletions of *bar, ap*-*3* and *prk*-*6* in selected 22 transformants with co-transformation of three donor-DNAs without CRISPR expressing cassettes. (A) Schematic of homologous recombination (HR) of target genes mediated by donor DNA. (B) PCR analysis of triple-gene deletion of *bar, ap*-*3* and *prk*-*6* in selected 22 transformants using one primer (alp1-out-F2, ap3/prk6-out-F) located upstream of the 5′ flanking region of genomic DNA and the other primer (alp1-in-R2, gh1-1/res1-in-R) located in the 3′ flanking region of genomic DNA. The expected lengths of disrupted transformants of bar, ap-3 and prk-6 were 0.8, 2.0 and 0.8 kb, respectively, while those of the host strain (rightmost lane) was 2.0, 1.2 and 1.2 kb, respectively. Heterokaryotic transformants showed two PCR bands (both of wild-type and knockout). HDR, homology-directed repair.

**Additional file 10: Figure S9.** Second round of target genomic editing by CRISPR–Cas9 system. (A) Schematic of homologous recombination (HR) of *neo*, *alp*-*1*, *rca*-*1* and *hcr*-*1* mediated by Cas12a, array2 and donor DNA. (B) PCR analysis of quadruple-gene deletion of *neo*, *alp*-*1*, *rca*-*1* and *hcr*-*1* in selected transformants using one primer (gh1-1-out-F2, alp1/rca1/hcr1-out-F) located upstream of the 5′ flanking region of genomic DNA and the other primer (gh1-1-in-R2, alp1/rca1/hcr1-in-R) located in the 3′ flanking region of genomic DNA. The expected lengths of disrupted transformants of neo, alp1, rca-1 and hcr-1 were 0.8, 1.6, 5.0 and 0.7 kb, respectively, while those of the host strain (rightmost lane) was 1.9, 1.0, 0.6 and 1.0 kb, respectively. Heterokaryotic transformants showed two PCR bands (both of wild-type and knockout). Symbol star indicated deletion mutant. HDR, homology-directed repair.

**Additional file 11: Figure S10.** Third round of target genomic editing by CRISPR–Cas9 system. (A) Schematic of homologous recombination (HR) of *bar*, *ap*-*3* and *prk*-*6* mediated by Cas12a, array2 and donor DNA. (B) PCR analysis of triple-gene deletion of *bar*, *ap*-*3* and *prk*-*6* in selected 22 transformants using one primer (alp1-out-F2, ap3/prk6-out-F) located upstream of the 5′ flanking region of genomic DNA and the other primer (alp1-in-R2, gh1-1/res1-in-R) located in the 3′ flanking region of genomic DNA. The expected lengths of disrupted transformants of *bar*, *ap3* and *prk6* were 0.8, 2.0 and 0.8 kb, respectively, while those of the host strain (rightmost lane) was 2.0, 1.2 and 1.2 kb, respectively. Heterokaryotic transformants showed two PCR bands (both of wild-type and knockout). The symbol of star indicated deletion mutant. HDR, homology-directed repair.

**Additional file 12: Figure S11.** SDS-PAGE of secreted protein from the eight mutant strains and wild type strain (WT) cultured for 6 days in Avicel inducing medium supplemented with 0.75% yeast extract.

**Additional file 13: Table S2.** List of Cas12a or Cas9 guide and PAM sequences in this study.

**Additional file 14:** Nucleotide sequence of the Cas12a expression cassette. Purple letters indicate the *tef1* (translation elongation factor EF-1, MYCTH_2298136) promoter P*tef1*. Blue letters indicate the nuclear localization signal. Red letters indicate the *Cas12a* gene. Gray letters indicate the T*trpC* terminator from A. nidulans *trpC* gene.

**Additional file 15.** Nucleotide sequence of the crRNA expression cassettes. Blue letters indicate the RNA polymerase III U6 snRNA promoter. Red letters indicate the 19 nt direct repeat. Green letters indicate the target sequence of *amdS*, *cre*-*1*, *res*-*1*, *gh1*-*1*, *alp*-*1*, *neo*, *rca*-*1*, *hcr*-*1*, *bar*, *ap*-*3*, or *prk*-*6*, respectively.

## Data Availability

All data generated or analyzed during this study are included in this published article and its supplementary information file.

## Abbreviations

CRISPR: clustered regularly interspaced short palindromic repeats
Camr technology: CRISPR–Cas-assisted marker recycling technology
HR: homologous recombination
sgRNA: single chimeric guide RNA
crRNA: CRISPR RNA
tracrRNA: a trans-activating CRISPR RNA
PAM: protospacer adjacent motif
DSB: double-strand break
NHEJ: nonhomologous end joining
HDR: homology-directed repair
FAA: fluoroacetamide
CCR: carbon catabolite repression
WT: wild type
