# Supplementary material for: Upgrading of efficient and scalable CRISPR–Cas-mediated technology for genetic engineering in thermophilic fungus Myceliophthora thermophila
Source: Biotechnol Biofuels. 2019 Dec 23;12:293. doi: 10.1186/s13068-019-1637-y (PMC6927189; doi:10.1186/s13068-019-1637-y)
Supplement: Supplementary file 1 — Additional file 1: Table S1. List of PCR primers used in this study. [file 13068_2019_1637_MOESM1_ESM.docx]

**Additional file 1: Table S1. List of PCR primers used in this study.**

| Construction of Cas12a expressing vectors | | |
| --- | --- | --- |
| Cloning of *Cas12a* | Cas12a-F | GCCAGTTTCGTTCTTCAGAAAGCTTATGCCTCCAAGGAAACGCGCGAAGACCGAGGACGA |
| Cloning of *Cas12a* | Cas12a-R | TGATTTCAGTAACGTTAAGTTTAATTAATCATCACTCATCTTCTGTTTTGGCACGTTTC |
| Cloning of *tef1* promoter | Ptef1-F1 | CCCGGCGCGCCGAATTCCCGGGGATCCGTTTAAACTCCTCCGAGGTTCGACATCAGGGTT |
| Cloning of *tef1* promoter | Ptef1-R1 | GGTCTTCGCGCGTTTCCTTGGAGGCATAAGCTTTCTGAAGAACGAAACTGGCGACTTGCGC |
| Cloning of *TtrpC* terminator | TtrpC-F | CAGAAGATGAGTGATGATTAATTAAACTTAACGTTACTGAAATCATCAAA |
| Cloning of *TtrpC* terminator | TtrpC-R | CTGCCCGTCACAGAGATTTGACCATGGGAGCTCCCTCTAAACAAGTGTACCTGTGCAT |
| Construction of crRNA expressing plasmids | | |
| Cloning U6p | U6p-F | AGGATCGGTGGAGTGAAGTTCGGAA |
| Cloning of cre1 crRNA | Cpf1cre1T-R | AAAAAATTGGAGTCTGCTCCCTTTCCGCCATCTACAAGAGTAGAAATTCGAGGAAAGAAAGAAAAGA |
| Cloning of res1 crRNA | Cpf1res1T-R | AAAAAACAGGCCGGAGCTGGGGCGAGGCAATCTACAAGAGTAGAAATTCGAGGAAAGAAAGAAAA |
| Cloning of gh1-1 crRNA | Cpf1gh1T-R | AAAAAAGAACATGACGCGCGCGTAGTTCTATCTACAAGAGTAGAAATTCGAGGAAAGAAAGAAAA |
| Cloning of array1 | array1-R | AAAAAAGAACATGACGCGCGCGTAGTTCTATCTACAAGAGTAGAAAT |
| Cloning of array2 | array2-R | AAAAAACTCGGGCACTTGTACCGACTCTGATCTACAAGAGTAGAAATT |
| Cloning of array3 | array3-R | AAAAAATGCTTCGGCTTGGGGTCTTGTTTATCTACAAGAGTAGAAATT |
| Construction of sgRNA expressing plasmids | | |
| Cloning U6p | U6p-F | AGGATCGGTGGAGTGAAGTTCGGAA |
| Cloning U6p | U6p-neo-R | CTAGCTCTAAAACATTGTCTGTTGTGCCCAGTCGAGGAAAGAAAGAAAAGAAGAGGAG |
| Cloning U6p | U6p-rca1-R | GCTCTAAAACCCGTCGACGGCGAGAGGTCCGAGGAAAGAAAGAAAAGAAGAGGAG |
| Cloning U6p | U6p-hcr1-R | GCTCTAAAACACTCTGGAAAGGGCGACGTCGAGGAAAGAAAGAAAAGAAGAGGAG |
| Cloning U6p | U6p-ap3-R | GCTCTAAAACAGGAATTCTTCGAGCGCATCGAGGAAAGAAAGAAAAGAAGAGGAG |
| Cloning U6p | U6p-bar-R | GCTCTAAAACTCCACTCCTGCGGTTCCTGCGAGGAAAGAAAGAAAAGAAGAGGAG |
| Cloning U6p | U6p-prk6-R | GCTCTAAAACACGCTCCACATGTCGATGGCGAGGAAAGAAAGAAAAGAAGAGGAG |
| *neo*-gRNA 5′ | g-neo-F | CTTTCTTTCCTCGACTGGGCACAACAGACAATGTTTTAGAGCTAGAAATAGCAAGTT |
| *rca1*-gRNA 5′ | g-rca1-F | TTCTTTCCTCGGACCTCTCGCCGTCGACGGGTTTTAGAGCTAGAAATAGCAAGTT |
| *hcr1*-gRNA 5′ | g-hcr1-F | TTCTTTCCTCGACGTCGCCCTTTCCAGAGTGTTTTAGAGCTAGAAATAGCAAGTT |
| *bar*-gRNA 5′ | g-bar-F | TTCTTTCCTCGCAGGAACCGCAGGAGTGGAGTTTTAGAGCTAGAAATAGCAAGTT |
| *ap3*-gRNA 5′ | g-ap3-F | TTCTTTCCTCGATGCGCTCGAAGAATTCCTGTTTTAGAGCTAGAAATAGCAAGTT |
| *bar*-gRNA 5′ | g-bar-F | TTCTTTCCTCGCAGGAACCGCAGGAGTGGAGTTTTAGAGCTAGAAATAGCAAGTT |
| *prk6*-gRNA 5′ | g-prk6-F | TTCTTTCCTCGCCATCGACATGTGGAGCGTGTTTTAGAGCTAGAAATAGCAAGTT |
| gRNA 3′ | gRNA-R | AAAAAAAGCACCGACTCGGTGCCACTT |
| Donor DNA construction | | |
| *cre1*-upstream | cre1-5′-F | TTGGGCCCGGCGCGCCGAATTCCCGGGGATCCACCGGGGTAAGGTACTCTGTAAGTT |
| *cre1*-upstream | cre1-5′-R | GCTCCTTCAATATCAGTTAACGTCGTATGTCCACCCTCCCAAGCGGAGTT |
| Cloning of PtrpC-neo | neo-cre1-F | AACTCCGCTTGGGAGGGTGGACATACGACGTTAACTGATATTGAAGGAGC |
| Cloning of PtrpC-neo | neo-cre1-R | AAGCGTTTGCTACACCCGGGGAACTTCAGAAGAACTCGTCAAGAAGGCGA |
| *cre1*-downstream | cre1-3′-F | TCGCCTTCTTGACGAGTTCTTCTGAAGTTCCCCGGGTGTAGCAAACGCTT |
| *cre1*-downstream | cre1-3′-R | CAGATCTACCATGGTGGACTCCTCTTAAAGCTTTGGGGTGTGGTCCGGCGTCGGGGAC |
| *cre1*-upstream | cre1-5′-F2 | ACAGGAAACAGCTATGACCATGATTACGAATTCACCGGGGTAAGGTACTCTGTA |
| *cre1*-upstream | cre1-5′-R2 | TTGGGATTGTTGTGTATCCTCGAGTTTATTATATGTCCACCCTCCCAAGCGGAGTT |
| *cre1*-downstream | cre1-3′-F2 | AACTCCGCTTGGGAGGGTGGACATATAATAAACTCGAGGATACACAACAATCCCAA |
| *cre1*-downstream | cre1-3′-R2 | CGACGTTGTAAAACGACGGCCAGTGCCAAGCTTCGTCGGGGACAACGAGTTGTGGGAA |
| *res1*-upstream | res1-5′-F | ACAGGAAACAGCTATGACCATGATTACGAATTCAACCAACCCTCCTTCGACTTTC |
| *res1*-upstream | res1-5′-R | AAGTGGGACTGGTACGAGACAAGACTCATCAGTCCGGGTGAATTAAGGAGGGAT |
| *res1*-downstream | res1-3′-F | ATCCCTCCTTAATTCACCCGGACTGATGAGTCTTGTCTCGTACCAGTCCCACTT |
| *res1*-downstream | res1-3′-R | CGACGTTGTAAAACGACGGCCAGTGCCAAGCTTGGTGCTCGTAAAAGTCTTGT |
| *gh1-1*-upstream | gh1-1-5′-F | TTGGGCCCGGCGCGCCGAATTCCCGGGGATCCACTATCAACAGCCCCCCGGATTGCT |
| *gh1-1*-upstream | gh1-1-5′-R | GCTCCTTCAATATCAGTTAACGTCGTGGAGGGGCCACGGCCATCCTCGTT |
| Cloning of PtrpC-neo | neo gh1-1-F | AACGAGGATGGCCGTGGCCCCTCCACGACGTTAACTGATATTGAAGGAGC |
| Cloning of PtrpC-neo | neo- gh1-1-R | TTGCACTTGGGGATGGCCTTGAACATCAGAAGAACTCGTCAAGAAGGCGA |
| *gh1-1*-downstream | gh1-1-3′-F | TCGCCTTCTTGACGAGTTCTTCTGATGTTCAAGGCCATCCCCAAGTGCAA |
| *gh1-1*-downstream | gh1-1-3′-R | CAGATCTACCATGGTGGACTCCTCTTAAAGCTTGCAGCCAGAAGGACTGCGTCTCGGG |
| *neo*-upstream | gh1-1-5′-F2 | ACAGGAAACAGCTATGACCATGATTACGAATTCCCCACTATCAACAGCCCCCCGGATT |
| *neo*-upstream | gh1-1-5′-R2 | TTGCACTTGGGGATGGCCTTGAACATTACTGGTACCTGTGGGTGAGGAGAGAAA |
| *neo*-downstream | gh1-1-3′-F2 | TTTCTCTCCTCACCCACAGGTACCAGTAATGTTCAAGGCCATCCCCAAGTGCAA |
| *neo*-downstream | gh1-1-3′-R2 | CGACGTTGTAAAACGACGGCCAGTGCCAAGCTTGCAGCCAGAAGGACTGCGT |
| *alp1*-upstream | alp1-5′-F1 | ACAGGAAACAGCTATGACCATGATTACGAATTCCCTTCCTAGTCCTCGCAGCAAACTC |
| *alp1*-upstream | alp1-5′-R1 | GCTCCTTCAATATCAGTTAACGTCGTAGACGTGGTCGGCCTTGGAGCGGA |
| Cloning PtrpC-bar | bar-alp1-F | TCCGCTCCAAGGCCGACCACGTCTACGACGTTAACTGATATTGAAGGAG |
| Cloning PtrpC-bar | bar-alp1-R | TGTCGTAGGTGTAGGTGGTGGAGCCTCAAATCTCGGTGACGGGCAGGACC |
| *alp1*-downstream | alp1-3′-F1 | GGTCCTGCCCGTCACCGAGATTTGAGGCTCCACCACCTACACCTACGACA |
| *alp1*-downstream | alp1-3′-R1 | CGACGTTGTAAAACGACGGCCAGTGCCAAGCTTAGTTGGCGGCGTTCTGGTTCTCGTT |
| *hcr1*-upstream | hcr1-5′-F | ACAGGAAACAGCTATGACCATGATTACGAATTCAATCCCCACCGGGAACCGGAACCTG |
| *hcr1*-upstream | hcr1-5′-R | TTGTCTTCCGGGGGAGCTGGGCGTTTTATTAAAGGGGCTTCTGAGATGCCAAGGCA |
| *hcr1*-downstream | hcr1-3′-F | TGCCTTGGCATCTCAGAAGCCCCTTTAATAAAACGCCCAGCTCCCCCGGAAGACAA |
| *hcr1*-downstream | hcr1-3′-R | CGACGTTGTAAAACGACGGCCAGTGCCAAGCTTGCGTTCTAGAGCCGGGGGTGTACAT |
| *rca1*-upstream | rca1-5′-F | ACAGGAAACAGCTATGACCATGATTACGAATTCCCGGCTCTCCACTTCACTTCCTAAA |
| *rca1*-upstream | rca1-5′-R | CCGCTGCCCCTGAACAAACTTTGGGATGAAACTGCTGCTGTTGTTGATGC |
| *rca1*-downstream | rca1-3′-F | TTCCCCCCCTTATAACCGGCGATGCCGCTTTCCGAGCGACGGGGGATCTT |
| *rca1*-downstream | rca1-3′-R | CGACGTTGTAAAACGACGGCCAGTGCCAAGCTTTGCGTGAAACCGTGTACACTGTCTC |
| Cloning of P*hsp70* | Phsp70-F | GCATCAACAACAGCAGCAGTTTCATCCCAAAGTTTGTTCAGGGGCAGCGG |
| Cloning of P*hsp70* | Phsp70-R | ACCGGTGAAGCGGGTTAGACAACATCTTGTGTGTTCGCAATCTGAAAGGG |
| Cloning of *xyr1* | Xyr1-F | CCCTTTCAGATTGCGAACACACAAGATGTTGTCTAACCCGCTTCACCGGT |
| Cloning of *xyr1* | Xyr1-R | AAGATCCCCCGTCGCTCGGAAAGCGGCATCGCCGGTTATAAGGGGGGGAA |
| *ap3*-upstream | ap3-5′-F | ACAGGAAACAGCTATGACCATGATTACGAATTCCGTTGGCGTGGTTCGAGAGACCGCA |
| *ap3*-upstream | ap3-5′-R | GCTCCTTCAATATCAGTTAACGTCGTTGGTGTTGGGCAGGTAGATGAGGT |
| Cloning PtrpC-neo | neo-ap3-F | ACCTCATCTACCTGCCCAACACCAACGACGTTAACTGATATTGAAGGAGC |
| Cloning PtrpC-neo | neo-ap3-R | TTGGGGCGGTGAACGTATTTGCGAATCAGAAGAACTCGTCAAGAAGGCGA |
| *ap3*-downstream | ap3-3′-F | TCGCCTTCTTGACGAGTTCTTCTGATTCGCAAATACGTTCACCGCCCCAA |
| *ap3*-downstream | ap3-3′-R | CGACGTTGTAAAACGACGGCCAGTGCCAAGCTTCGACACCAGCTCCTCCTCCCCTCAA |
| *bar*-upstream | alp1-5′-F2 | ACAGGAAACAGCTATGACCATGATTACGAATTCTGTCTTGCTTTCTGGCCTGCCCTTT |
| *bar*-upstream | alp1-5′-R2 | TTGGTCTTCTTGGCAACACCGTACGTTAGTCGCGGAGCTTGATGATGTACTT |
| *bar*-downstream | alp1-3′-F2 | AAGTACATCATCAAGCTCCGCGACTAACGTACGGTGTTGCCAAGAAGACCAA |
| *bar*-downstream | alp1-3′-R2 | CGACGTTGTAAAACGACGGCCAGTGCCAAGCTTGGAGAAATAAATACGGGGCGGGAAT |
| *prk6*-upstream | prk6-5′-F | ACAGGAAACAGCTATGACCATGATTACGAATTCGCAGCACAAGCACAAGCACAAGCAG |
| *prk6*-upstream | prk6-5′-R | GTCGGGACGCCGCAGAGCTCGAAGATTAGGGCATGTCTTCGAGGATGGATT |
| *prk6*-downstream | prk6-3′-F | AATCCATCCTCGAAGACATGCCCTAATCTTCGAGCTCTGCGGCGTCCCGAC |
| *prk6*-downstream | prk6-3′-R | CGACGTTGTAAAACGACGGCCAGTGCCAAGCTTCAGCATCTTTTGCATCATCTCCGCG |
| PCR analysis of point mutations and gene deletion | | |
| Sequencing and verifying *amdS* | amdS-in-F | ATGCCTCAATCCTGGGAAGAACTGG |
| Sequencing and verifying *amdS* | amdS-in-R | CCCTTCGTCGTACTTGTTTAGCCAT |
| PCR detecting *cre1* disruption | cre1-out-F | ATACAGTACCTCTGCACAACCATCC |
| PCR detecting *cre1* disruption | cre1-in-R1 | AGTTGGGATTGTTGTGTATCCTCGA |
| PCR detecting *cre1* disruption | cre1-in-R2 | TTGGGTGAAGACAATGCCGTGGGGG |
| PCR detecting res1 disruption | res1-out-F | AACCCCAGGCATCGTAGATCAGGGC |
| PCR detecting res1 disruption | res1-in-R | GAGAAGTGGGACTGGTACGAGACAA |
| PCR detecting *gh1-1* disruption | gh1-1-out-F | CCTCAGTCTCCGCAATATCGTGCAT |
| PCR detecting *gh1-1* disruption | gh1-1-in-R1 | GAAGTAGCCCGTGTTGTAGCCCAGG |
| PCR detecting *gh1-1* disruption | gh1-1-in-R2 | GCGATGAGGATGTTGTGGCCGACGA |
| PCR detecting *alp1* disruption | alp1-out-F1 | TTCTGGCCTGCCCTTTTCTTTCAAC |
| PCR detecting *alp1* disruption | alp1-in-R1 | GTAGATGCCCGTGTCGATCACATAG |
| PCR detecting *alp1* disruption | alp1-out-F2 | CAAGCACTTGATCACGGCGGGAGCA |
| PCR detecting *alp1* disruption | alp1-in-R2 | TCAGTCAGAAGATAACGGCCGGGCG |
| PCR detecting *rca1* disruption | rca1-out-F | CGCAGACACATCCTCAACCCCGGCT |
| PCR detecting *rca1* disruption | rca1-in-R | AAGATCCCCCGTCGCTCGGAAAGCG |
| PCR detecting *hcr1* disruption | hcr1-out-F | CCCTCACAGTACGGCCTGTCCCTGT |
| PCR detecting *hcr1* disruption | hcr1-in-R | AACAAGCATCGCCGTCAGCGTCAGT |
| PCR detecting *ap3* disruption | ap3-out-F | TGAGGCACTCACCGACGATTCTGCA |
| PCR detecting *ap3* disruption | ap3-in-R | CGATCACGTCGCAGTAGAGCTTGTT |
| PCR detecting *prk6* disruption | prk6-out-F | AGCAGAGAAGCAAGCTCGGGAAGCT |
| PCR detecting *prk6* disruption | prk6-in-R | GCATCTCTTTCGCGGTCGGCCTCTT |
