# Supplementary material for: Upgrading of efficient and scalable CRISPR–Cas-mediated technology for genetic engineering in thermophilic fungus Myceliophthora thermophila
Source: Biotechnol Biofuels. 2019 Dec 23;12:293. doi: 10.1186/s13068-019-1637-y (PMC6927189; doi:10.1186/s13068-019-1637-y)
Supplement: Supplementary file 2 — Additional file 2: Figure S1. Verification of cre-1 gene deletions in selected transformants with co-transformation of only Cas12a and donor DNA, only crRNA and donor DNA, or only donor DNA. (A) Schematic of homologous recombination (HR) of target gene cre-1. (B–D) PCR analysis of cre-1 deletion with one primer (cre1-out-F) located upstream of the 5′ flanking region of genomic DNA and the other (cre1-in-R) located in the 3′ flanking region of genomic DNA. The expected length of disrupted transformants was 1.9 kb, while that of the WT host strain, used as a negative control, was 1.0 kb (rightmost lane). Heterokaryotic transformants showed two PCR bands (both of wild-type and knockout). HDR, homology-directed repair; WT, wild type. [file 13068_2019_1637_MOESM2_ESM.pdf]

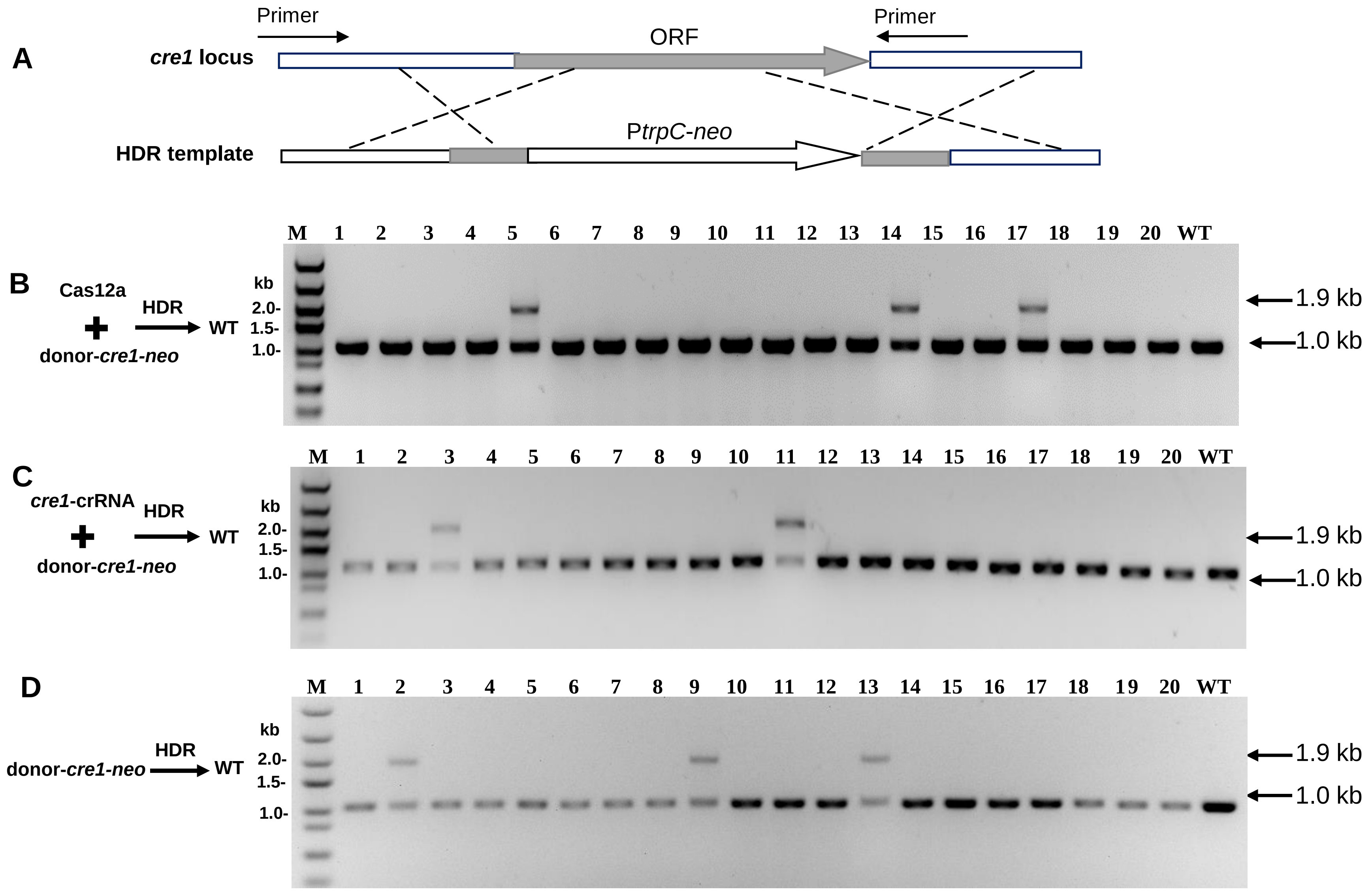

Figure S1 Verification of *cre-1* gene deletions in selected transformants with co-transformation of only Cas12a and donor DNA, only crRNA and donor DNA, or only donor DNA. (A) Schematic of homologous recombination (HR) of target gene *cre-1*. (B–D) PCR analysis of *cre-1* deletion (B–D) with one primer (cre1-out-F) located upstream of the 5′ flanking region of genomic DNA and the other (cre1-in-R) located in the 3′ flanking region of genomic DNA. The expected length of disrupted transformants was 1.9 kb, while that of the WT host strain, used as a negative control, was 1.0 kb (rightmost lane). Heterokaryotic transformants showed two PCR bands (both of wild-type and knockout). HDR, homology-directed repair; WT, wild type.
