# Supplementary material for: Upgrading of efficient and scalable CRISPR–Cas-mediated technology for genetic engineering in thermophilic fungus Myceliophthora thermophila
Source: Biotechnol Biofuels. 2019 Dec 23;12:293. doi: 10.1186/s13068-019-1637-y (PMC6927189; doi:10.1186/s13068-019-1637-y)
Supplement: Supplementary file 4 — Additional file 4: Figure S3. Verification of triple-gene deletions of gh1-1, cre-1 and res-1 in selected transformants with co-transformation of only three donor-DNAs without CRISPR expressing cassettes. (A) Schematic of homologous recombination (HR) of target genes mediated by each donor DNA. (B) PCR analysis of triple-gene deletion of gh1-1, cre-1 and res-1 in selected 23 transformants using one primer (cre1/gh1-1/res1-out-F) located upstream of the 5′ flanking region of genomic DNA and the other primer (cre1/gh1-1/res1-in-R) located in the 3′ flanking region of genomic DNA. The expected lengths of disrupted transformants of gh1-1, cre-1 and res-1 were 1.9, 0.8 and 0.7 kb, respectively, while those of the host strain (rightmost lane) was 1.0, 1.2 and 0.9 kb, respectively. Heterokaryotic transformants showed two PCR bands (both of wild-type and knockout). [file 13068_2019_1637_MOESM4_ESM.pdf]

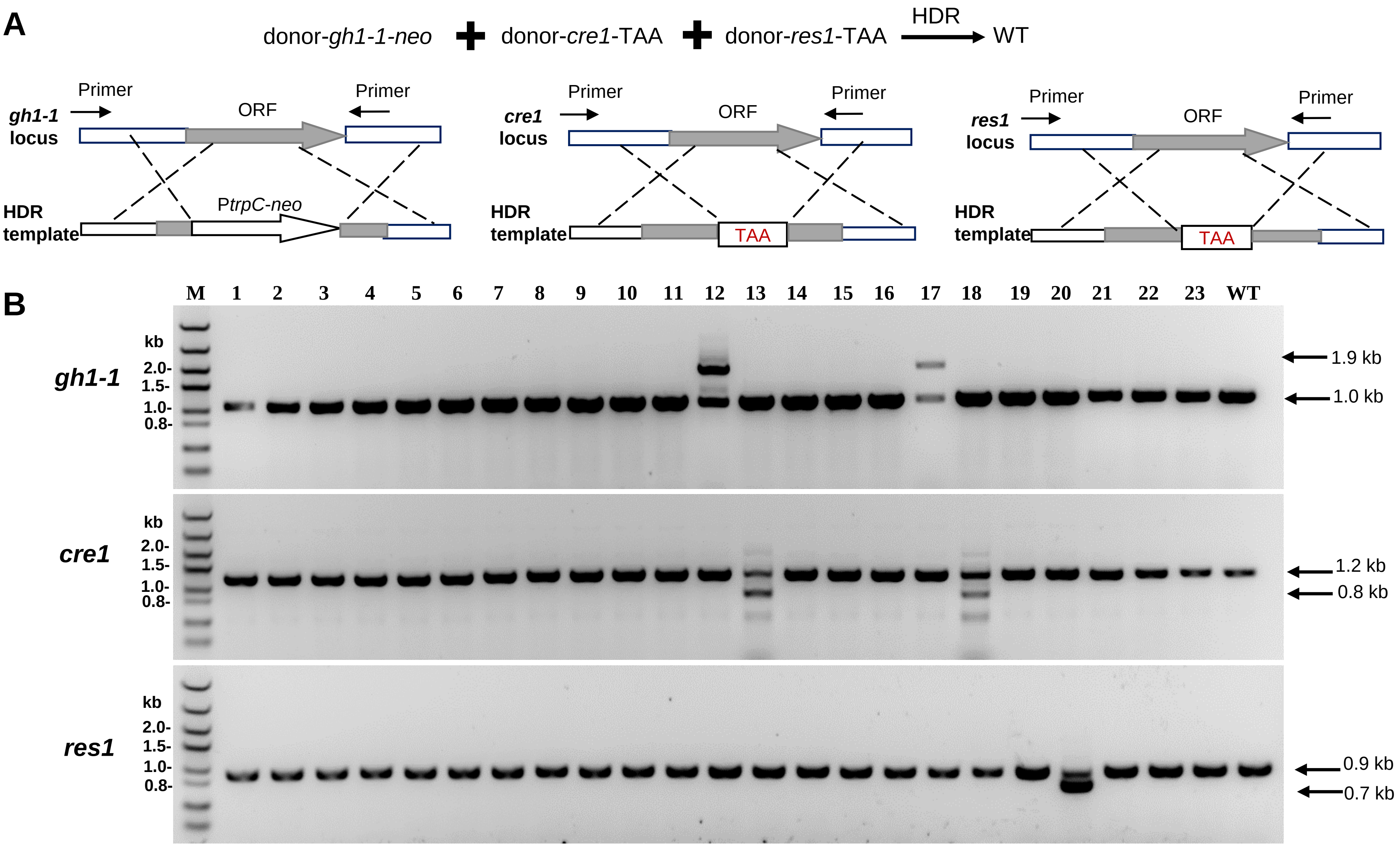

Figure S3 Verification of triple-gene deletions of *gh1-1*, *cre-1* and *res-1* in selected transformants with co-transformation of only three donor-DNAs without CRISPR expressing cassettes. (A) Schematic of homologous recombination (HR) of target genes mediated by each donor DNA. (B) PCR analysis of triple-gene deletion of *gh1-1*, *cre-1* and *res-1* in selected 23 transformants using one primer (cre1/gh1-1/res1-out-F) located upstream of the 5′ flanking region of genomic DNA and the other primer (cre1/gh1-1/res1-in-R) located in the 3′ flanking region of genomic DNA. The expected lengths of disrupted transformants of *gh1-1*, *cre-1* and *res-1* were 1.9, 0.8 and 0.7 kb, respectively, while those of the host strain (rightmost lane) was 1.0, 1.2 and 0.9 kb, respectively.
