# Supplementary material for: Upgrading of efficient and scalable CRISPR–Cas-mediated technology for genetic engineering in thermophilic fungus Myceliophthora thermophila
Source: Biotechnol Biofuels. 2019 Dec 23;12:293. doi: 10.1186/s13068-019-1637-y (PMC6927189; doi:10.1186/s13068-019-1637-y)
Supplement: Supplementary file 5 — Additional file 5: Figure S4. First round of target genomic editing by CRISPR–Cas9 system. (A) Schematic of homologous recombination (HR) of cre-1, res-1 and gh1-1 mediated by Cas9, sgRNAs and donor DNA. (B) PCR analysis of triple-gene deletion of cre-1, res-1 and gh1-1 in selected transformants using one primer (cre1/res1/gh1-1-out-F) located upstream of the 5′ flanking region of genomic DNA and the other primer (cre1/res1/gh1-1-in-R) located in the 3′ flanking region of genomic DNA. The expected lengths of disrupted transformants of cre-1, res-1 and gh1-1 were 0.8, 0.7 and 1.9 kb, respectively, while those of WT strain (rightmost lane) was 1.2, 0.9 and 1.0 kb, respectively. Heterokaryotic transformants showed two PCR bands (both of wild-type and knockout). The symbol of star indicated deletion mutant. HDR, homology-directed repair; WT, wild type. [file 13068_2019_1637_MOESM5_ESM.pdf]

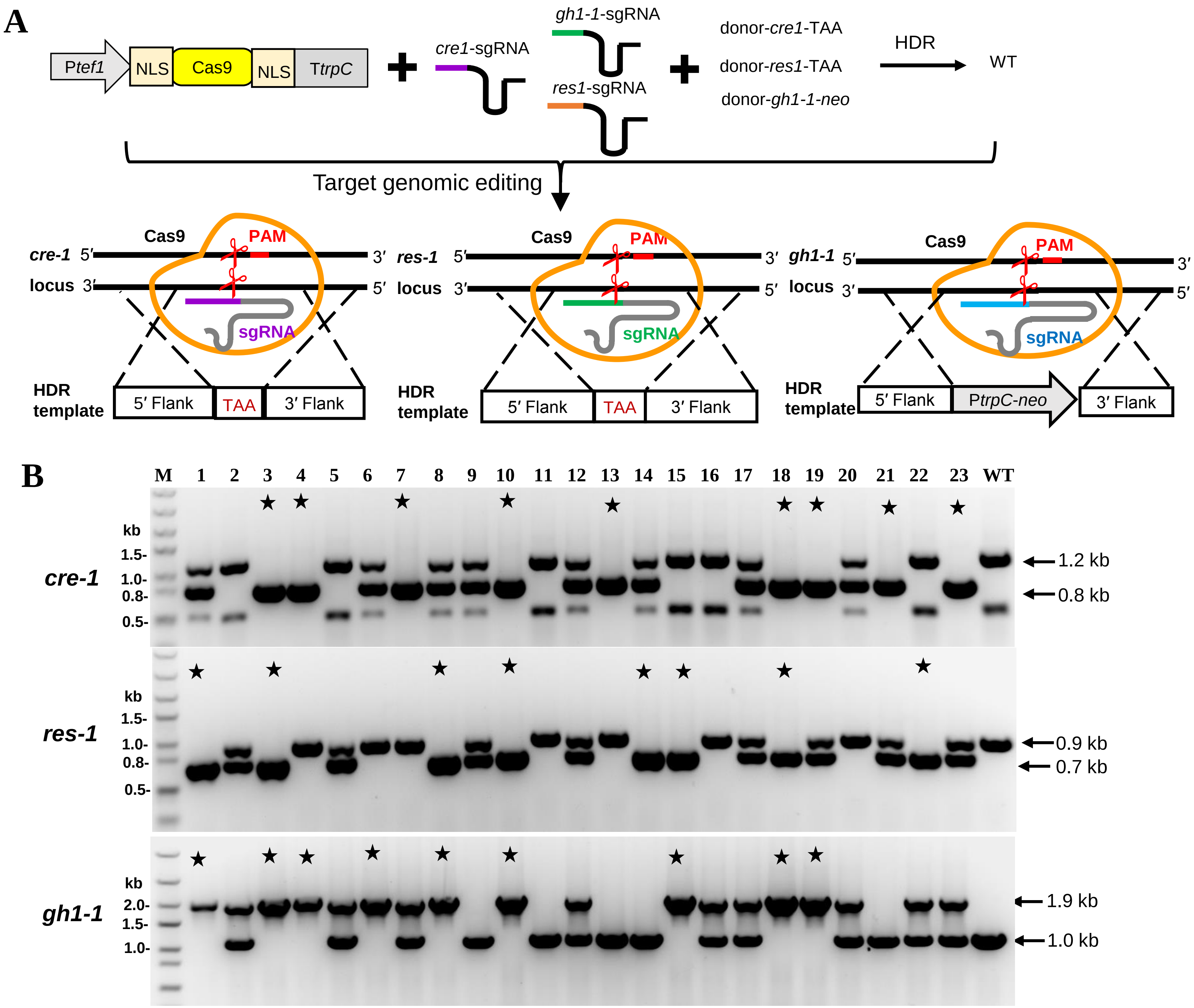

Figure S4. First round of target genomic editing by CRISPR-Cas9 system. (A) Schematic of homologous recombination (HR) of *cre-1*, *res-1* and *gh1-1* mediated by Cas9, sgRNAs and donor DNA. (B) PCR analysis of triple-gene deletion of *cre-1*, *res-1* and *gh1-1* in selected transformants using one primer (cre1/res1/gh1-1-out-F) located upstream of the 5′ flanking region of genomic DNA and the other primer (cre1/res1/gh1-1-in-R) located in the 3′ flanking region of genomic DNA. The expected lengths of disrupted transformants of *cre-1*, *res-1* and *gh1-1* were 0.8, 0.7 and 1.9 kb, respectively, while those of WT strain (rightmost lane) was 1.2, 0.9 and 1.0 kb, respectively. Heterokaryotic transformants showed two PCR bands (both of wild-type and knockout). The symbol of star indicated deletion mutant. HDR, homology-directed repair; WT, wild type.
