# Supplementary material for: Upgrading of efficient and scalable CRISPR–Cas-mediated technology for genetic engineering in thermophilic fungus Myceliophthora thermophila
Source: Biotechnol Biofuels. 2019 Dec 23;12:293. doi: 10.1186/s13068-019-1637-y (PMC6927189; doi:10.1186/s13068-019-1637-y)
Supplement: Supplementary file 6 — Additional file 6: Figure S5. Second round of target genomic editing by CRISPR–Cas12a system. (A) Schematic of homologous recombination (HR) of neo, alp-1, rca-1 and hcr-1 mediated by Cas12a, array2 and donor DNA. (B) PCR analysis of quadruple-gene deletion of neo, alp-1, rca-1 and hcr-1 in selected transformants using one primer (gh1-1-out-F2, alp1/rca1/hcr1-out-F) located upstream of the 5′ flanking region of genomic DNA and the other primer (gh1-1-in-R2, alp1/rca1/hcr1-in-R) located in the 3′ flanking region of genomic DNA. The expected lengths of disrupted transformants of neo, alp1, rca-1 and hcr-1 were 0.8, 1.6, 5.0 and 0.7 kb, respectively, while those of the host strain (rightmost lane) was 1.9, 1.0, 0.6 and 1.0 kb, respectively. Heterokaryotic transformants showed two PCR bands (both of wild-type and knockout). The symbol of star indicated deletion mutant. HDR, homology-directed repair; symbol star indicated deletion mutant. [file 13068_2019_1637_MOESM6_ESM.pdf]

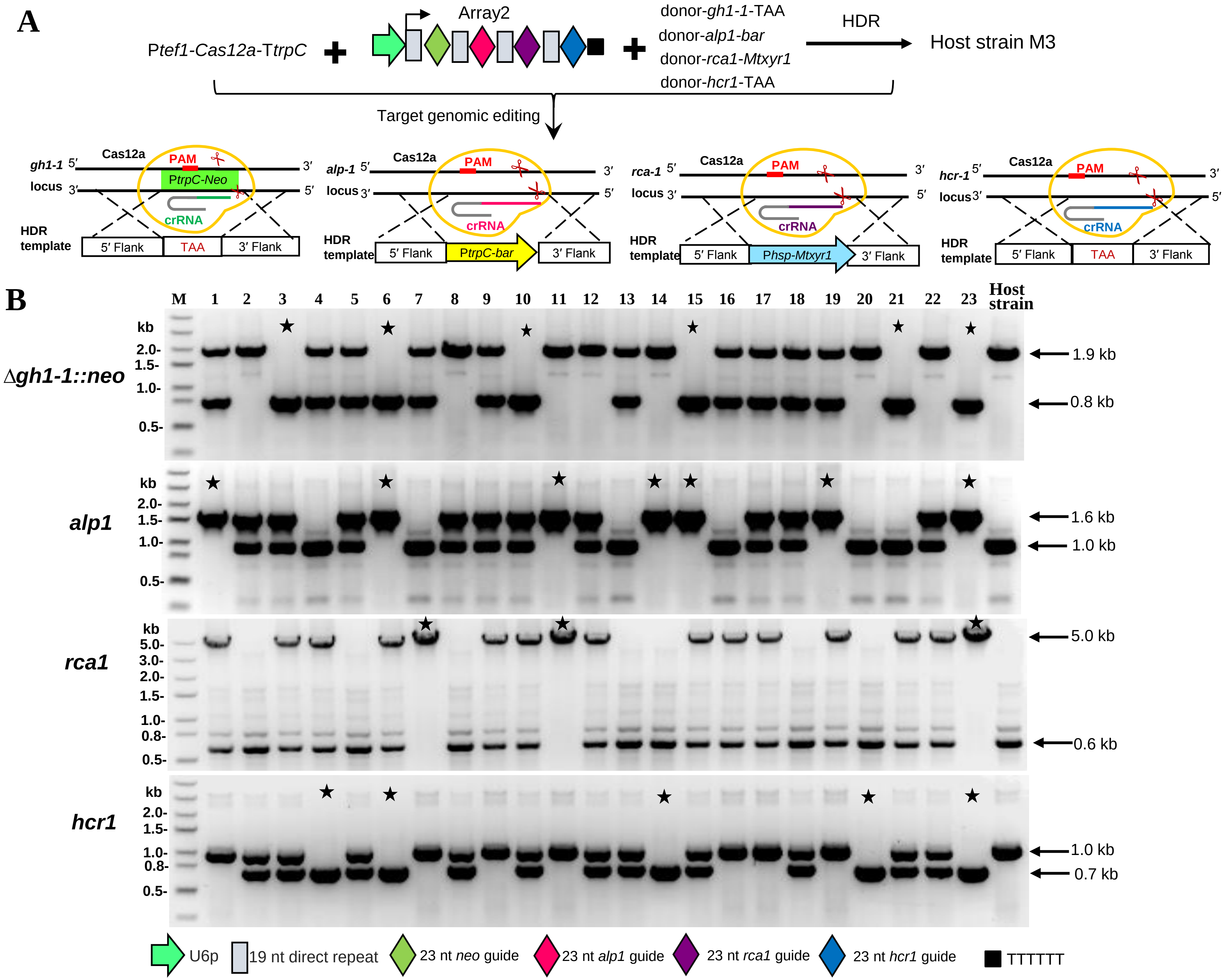

Figure S5. Second round of target genomic editing by CRISPR-Cas12a system. (A) Schematic of homologous recombination (HR) of *neo*, *alp-1*, *rca-1* and *hcr-1* mediated by Cas12a, array2 and donor DNA. (B) PCR analysis of quadruple-gene deletion of *neo*, *alp-1*, *rca-1* and *hcr-1* in selected transformants using one primer (gh1-1-out-F2, alp1/rca1/hcr1-out-F) located upstream of the 5′ flanking region of genomic DNA and the other primer (gh1-1-in-R2, alp1/rca1/hcr1-in-R) located in the 3′ flanking region of genomic DNA. The expected lengths of disrupted transformants of *neo*, *alp1*, *rca-1* and *hcr-1* were 0.8, 1.6, 5.0 and 0.7 kb, respectively, while those of the host strain (rightmost lane) was 1.9, 1.0, 0.6 and 1.0 kb, respectively. Heterokaryotic transformants showed two PCR bands (both of wild-type and knockout). The symbol of star indicated deletion mutant. HDR, homology-directed repair; symbol star indicated deletion mutant.
