# Supplementary material for: Upgrading of efficient and scalable CRISPR–Cas-mediated technology for genetic engineering in thermophilic fungus Myceliophthora thermophila
Source: Biotechnol Biofuels. 2019 Dec 23;12:293. doi: 10.1186/s13068-019-1637-y (PMC6927189; doi:10.1186/s13068-019-1637-y)
Supplement: Supplementary file 7 — Additional file 7: Figure S6. Verification of quadruple-gene deletions of neo, alp-1, rca-1 and hcr-1 in selected transformants with co-transformation of only four donor-DNAs without CRISPR expressing cassettes. (A) Schematic of homologous recombination (HR) of target genes mediated by each donor DNA. (B) PCR analysis of quadruple-gene deletion of neo, alp-1, rca-1 and hcr-1 in selected transformants using one primer (gh1-1-out-F2, alp1/rca1/hcr1-out-F) located upstream of the 5′ flanking region of genomic DNA and the other primer (gh1-1-in-R2, alp1/rca1/hcr1-in-R) located in the 3′ flanking region of genomic DNA. The expected lengths of disrupted transformants of neo, alp1, rca-1 and hcr-1 were 0.8, 1.6, 5.0 and 0.7 kb, respectively, while those of the host strain (rightmost lane) was 1.9, 1.0, 0.6 and 1.0 kb, respectively. Heterokaryotic transformants showed two PCR bands (both of wild-type and knockout). [file 13068_2019_1637_MOESM7_ESM.pdf]

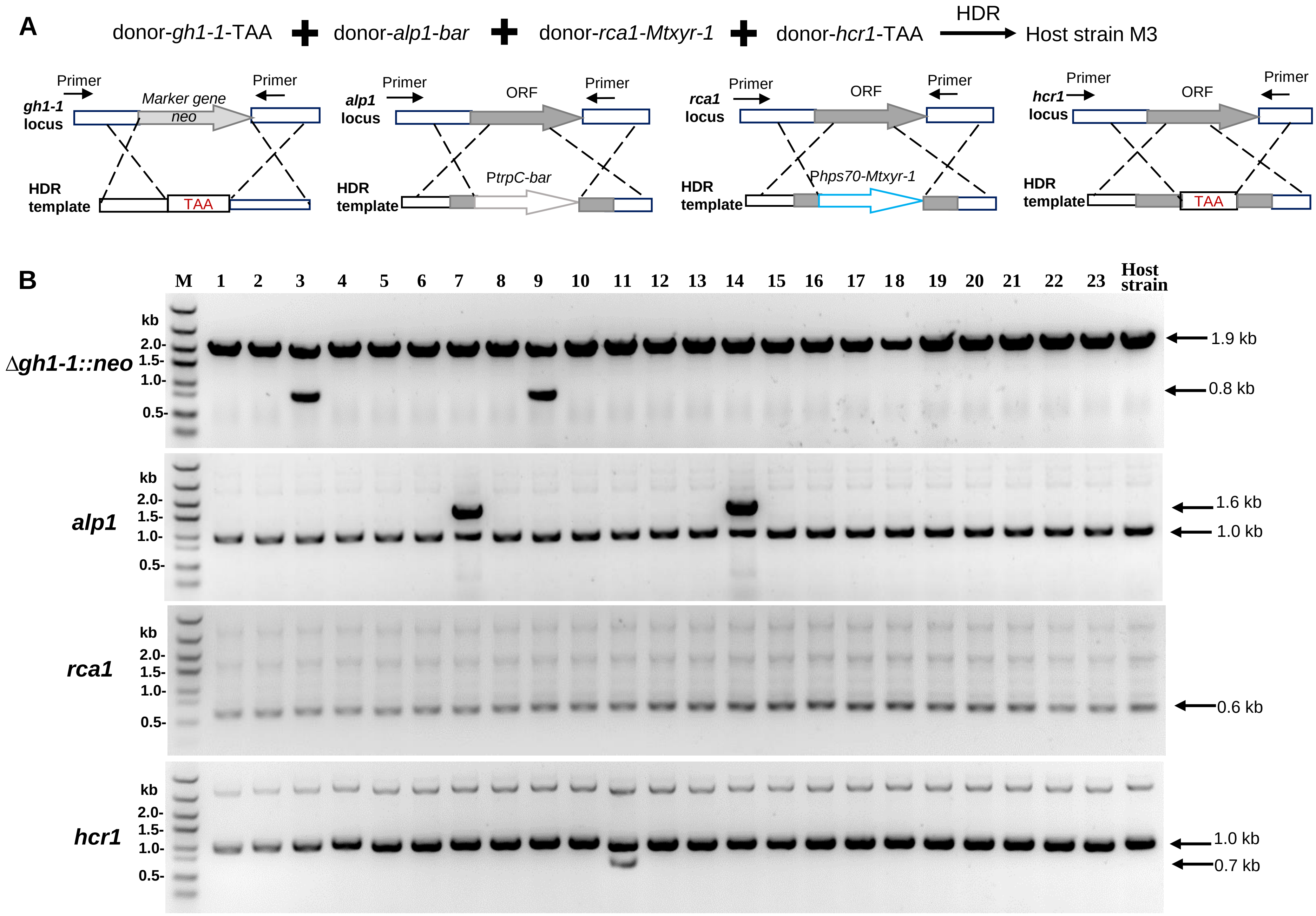

Figure S6 Verification of quadruple-gene deletions of *neo*, *alp-1*, *rca-1* and *hcr-1* in selected transformants with co-transformation of only four donor-DNAs without CRISPR expressing cassettes. (A) Schematic of homologous recombination (HR) of target genes mediated by each donor DNA. (B) PCR analysis of quadruple-gene deletion of *neo*, *alp-1*, *rca-1* and *hcr-1* in selected transformants using one primer (gh1-1-out-F2, alp1/rca1/hcr1-out-F) located upstream of the 5′ flanking region of genomic DNA and the other primer (gh1-1-in-R2, alp1/rca1/hcr1-in-R) located in the 3′ flanking region of genomic DNA. The expected lengths of disrupted transformants of *neo*, *alp1*, *rca-1* and *hcr-1* were 0.8, 1.6, 5.0 and 0.7 kb, respectively, while those of the host strain (rightmost lane) was 1.9, 1.0, 0.6 and 1.0 kb, respectively. Heterokaryotic transformants showed two PCR bands (both of wild-type and knockout).
