# Supplementary material for: Upgrading of efficient and scalable CRISPR–Cas-mediated technology for genetic engineering in thermophilic fungus Myceliophthora thermophila
Source: Biotechnol Biofuels. 2019 Dec 23;12:293. doi: 10.1186/s13068-019-1637-y (PMC6927189; doi:10.1186/s13068-019-1637-y)
Supplement: Supplementary file 8 — Additional file 8: Figure S7. Third round of target genomic editing by CRISPR–Cas12a system. (A) Schematic of homologous recombination (HR) of bar, ap-3 and prk-6 mediated by Cas12a, array2 and donor DNA. (B) PCR analysis of triple-gene deletion of bar, ap3 and prk6 in selected 22 transformants using one primer (alp1-out-F2, ap3/prk6-out-F) located upstream of the 5′ flanking region of genomic DNA and the other primer (alp1-in-R2, gh1-1/res1-in-R) located in the 3′ flanking region of genomic DNA. The expected lengths of disrupted transformants of bar, ap-3 and prk-6 were 0.8, 2.0 and 0.8 kb, respectively, while those of the host strain (rightmost lane) was 2.0, 1.2 and 1.2 kb, respectively. Heterokaryotic transformants showed two PCR bands (both of wild-type and knockout). The symbol of star indicated deletion mutant. HDR, homology-directed repair. [file 13068_2019_1637_MOESM8_ESM.pdf]

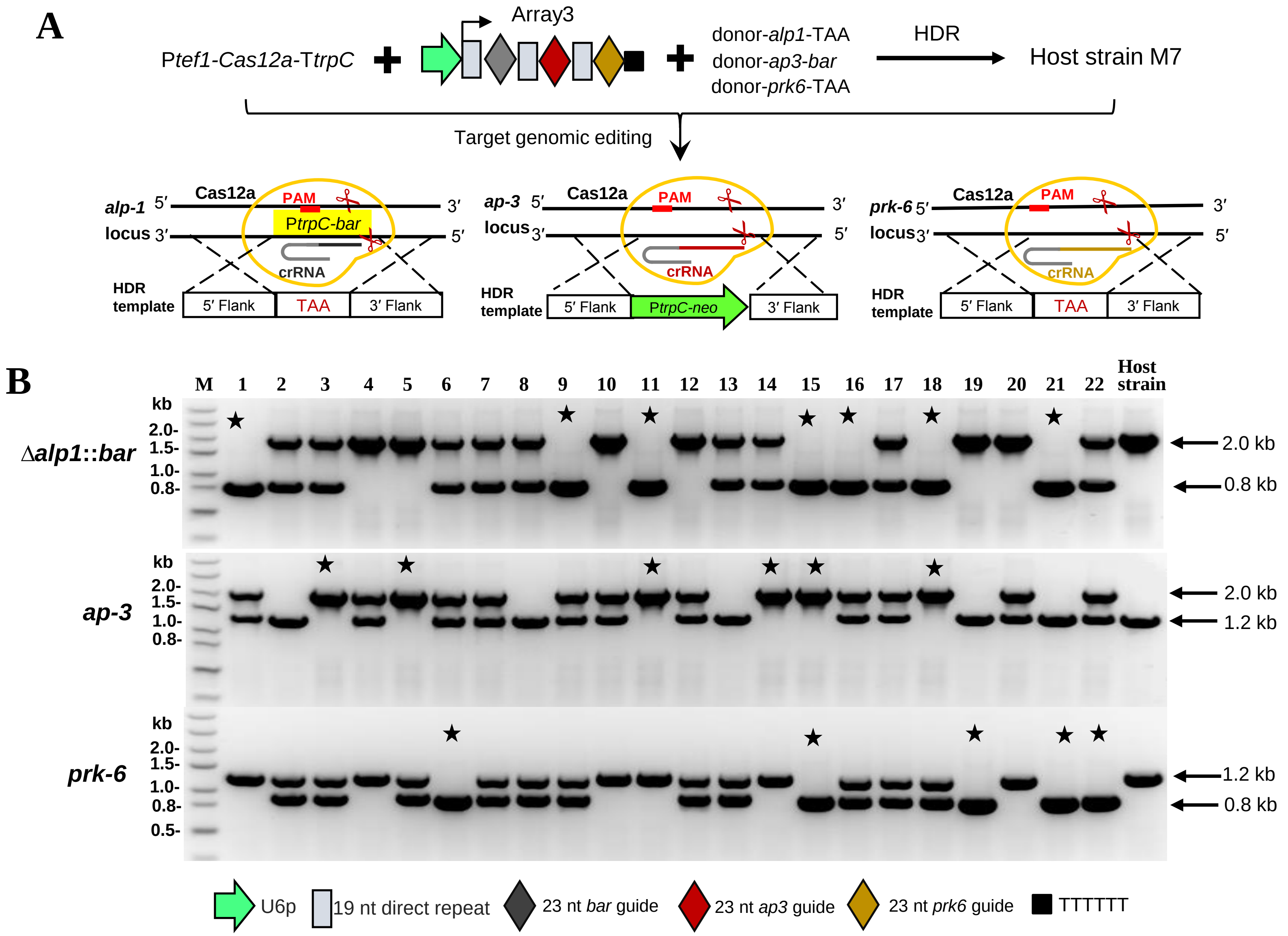

Figure S7. Third round of target genomic editing by CRISPR-Cas12a system. (A) Schematic of homologous recombination (HR) of *bar*, *ap-3* and *prk-6* mediated by Cas12a, array2 and donor DNA. (B) PCR analysis of triple-gene deletion of bar, ap3 and prk6 in selected 22 transformants using one primer (alp1-out-F2, ap3/prk6-out-F) located upstream of the 5′ flanking region of genomic DNA and the other primer (alp1-in-R2, gh1-1/res1-in-R) located in the 3′ flanking region of genomic DNA. The expected lengths of disrupted transformants of bar, ap3 and prk6 were 0.8, 2.0 and 0.8 kb, respectively, while those of the host strain (rightmost lane) was 2.0, 1.2 and 1.2 kb, respectively. Heterokaryotic transformants showed two PCR bands (both of wild-type and knockout). The symbol of star indicated deletion mutant. HDR, homology-directed repair.
