# Supplementary material for: Upgrading of efficient and scalable CRISPR–Cas-mediated technology for genetic engineering in thermophilic fungus Myceliophthora thermophila
Source: Biotechnol Biofuels. 2019 Dec 23;12:293. doi: 10.1186/s13068-019-1637-y (PMC6927189; doi:10.1186/s13068-019-1637-y)
Supplement: Supplementary file 9 — Additional file 9: Figure S8. Verification of triple-gene deletions of bar, ap-3 and prk-6 in selected 22 transformants with co-transformation of three donor-DNAs without CRISPR expressing cassettes. (A) Schematic of homologous recombination (HR) of target genes mediated by donor DNA. (B) PCR analysis of triple-gene deletion of bar, ap-3 and prk-6 in selected 22 transformants using one primer (alp1-out-F2, ap3/prk6-out-F) located upstream of the 5′ flanking region of genomic DNA and the other primer (alp1-in-R2, gh1-1/res1-in-R) located in the 3′ flanking region of genomic DNA. The expected lengths of disrupted transformants of bar, ap-3 and prk-6 were 0.8, 2.0 and 0.8 kb, respectively, while those of the host strain (rightmost lane) was 2.0, 1.2 and 1.2 kb, respectively. Heterokaryotic transformants showed two PCR bands (both of wild-type and knockout). HDR, homology-directed repair. [file 13068_2019_1637_MOESM9_ESM.pdf]

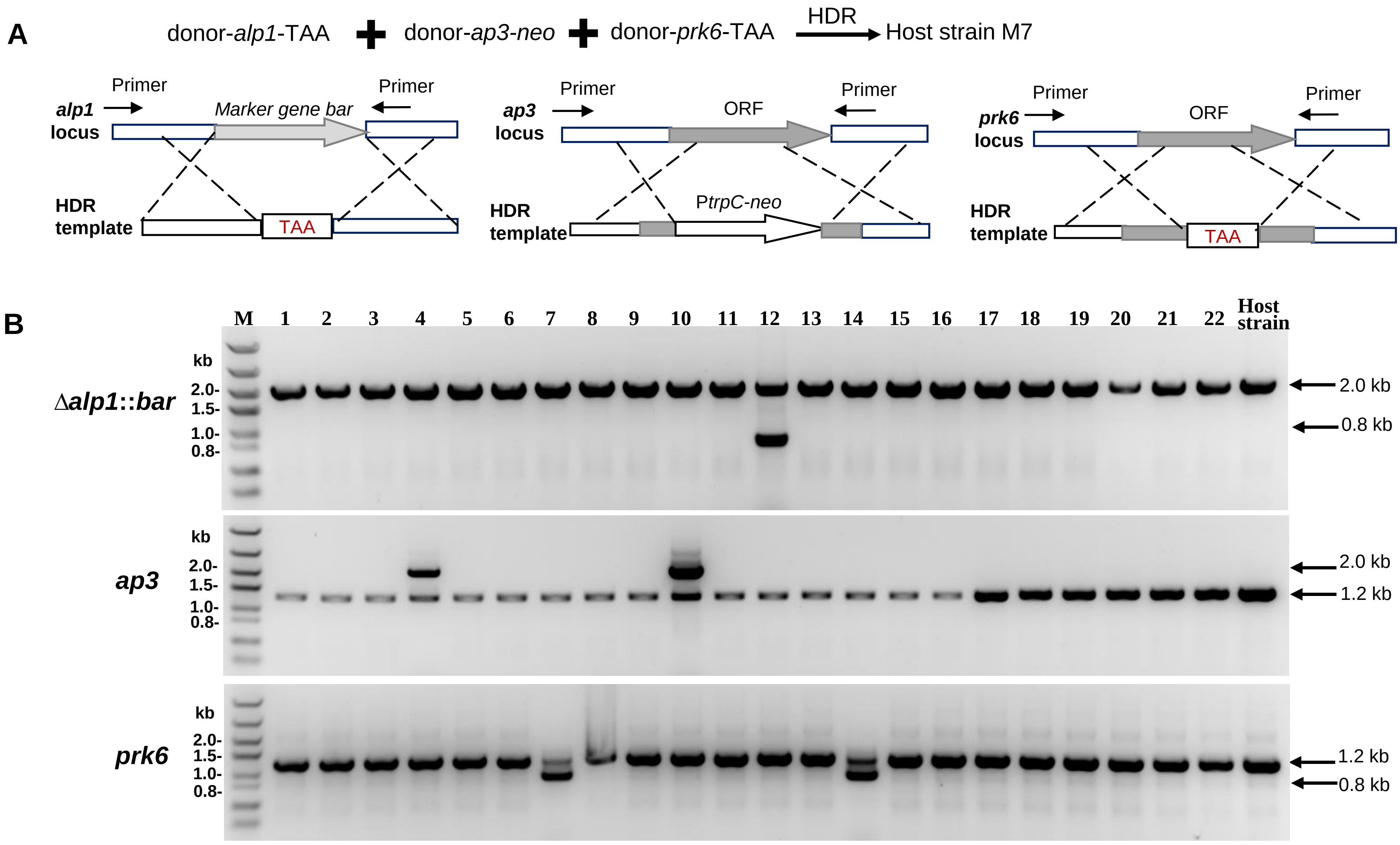

Figure S8 Verification of triple-gene deletions of *bar*, *ap-3* and *prk-6* in selected 22 transformants with co-transformation of three donor-DNAs without CRISPR expressing cassettes. (A) Schematic of homologous recombination (HR) of target genes mediated by donor DNA. (B) PCR analysis of triple-gene deletion of *bar*, *ap-3* and *prk-6* in selected 22 transformants using one primer (alp1-out-F2, ap3/prk6-out-F) located upstream of the 5′ flanking region of genomic DNA and the other primer (alp1-in-R2, gh1-1/res1-in-R) located in the 3′ flanking region of genomic DNA. The expected lengths of disrupted transformants of *bar*, *ap-3* and *prk-6* were 0.8, 2.0 and 0.8 kb, respectively, while those of the host strain (rightmost lane) was 2.0, 1.2 and 1.2 kb, respectively. Heterokaryotic transformants showed two PCR bands (both of wild-type and knockout). HDR, homology-directed repair.
