# Supplementary material for: Upgrading of efficient and scalable CRISPR–Cas-mediated technology for genetic engineering in thermophilic fungus Myceliophthora thermophila
Source: Biotechnol Biofuels. 2019 Dec 23;12:293. doi: 10.1186/s13068-019-1637-y (PMC6927189; doi:10.1186/s13068-019-1637-y)
Supplement: Supplementary file 10 — Additional file 10: Figure S9. Second round of target genomic editing by CRISPR–Cas9 system. (A) Schematic of homologous recombination (HR) of neo, alp-1, rca-1 and hcr-1 mediated by Cas12a, array2 and donor DNA. (B) PCR analysis of quadruple-gene deletion of neo, alp-1, rca-1 and hcr-1 in selected transformants using one primer (gh1-1-out-F2, alp1/rca1/hcr1-out-F) located upstream of the 5′ flanking region of genomic DNA and the other primer (gh1-1-in-R2, alp1/rca1/hcr1-in-R) located in the 3′ flanking region of genomic DNA. The expected lengths of disrupted transformants of neo, alp1, rca-1 and hcr-1 were 0.8, 1.6, 5.0 and 0.7 kb, respectively, while those of the host strain (rightmost lane) was 1.9, 1.0, 0.6 and 1.0 kb, respectively. Heterokaryotic transformants showed two PCR bands (both of wild-type and knockout). Symbol star indicated deletion mutant. HDR, homology-directed repair. [file 13068_2019_1637_MOESM10_ESM.pdf]

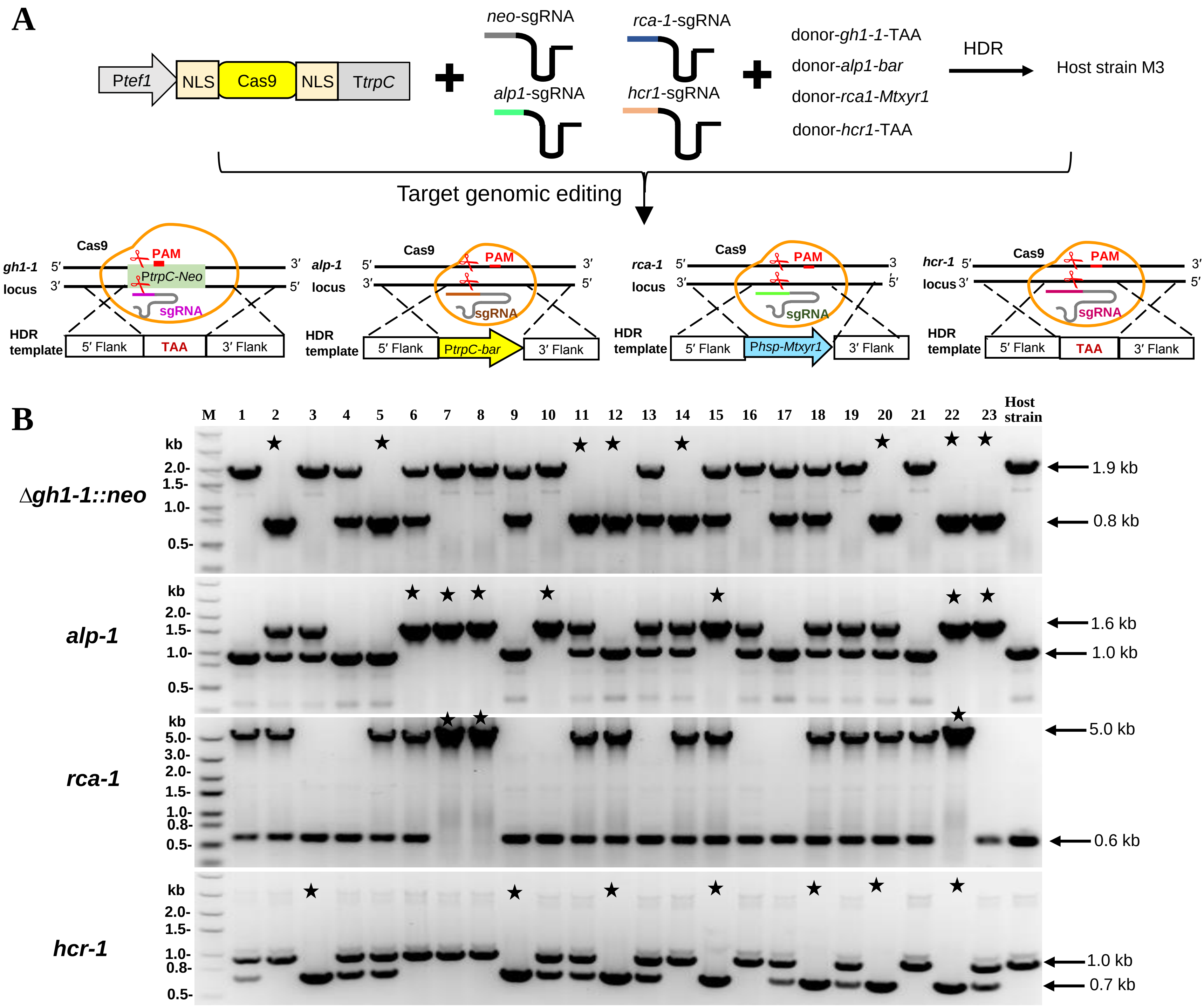

Figure S9. Second round of target genomic editing by CRISPR-Cas9 system. (A) Schematic of homologous recombination (HR) of *neo*, *alp-1*, *rca-1* and *hcr-1* mediated by Cas12a, array2 and donor DNA. (B) PCR analysis of quadruple-gene deletion of neo, alp-1, rca-1 and hcr-1 in selected transformants using one primer (gh1-1-out-F2, alp1/rca1/hcr1-out-F) located upstream of the 5′ flanking region of genomic DNA and the other primer (gh1-1-in-R2, alp1/rca1/hcr1-in-R) located in the 3′ flanking region of genomic DNA. The expected lengths of disrupted transformants of neo, alp1, rca-1 and hcr-1 were 0.8, 1.6, 5.0 and 0.7 kb, respectively, while those of the host strain (rightmost lane) was 1.9, 1.0, 0.6 and 1.0 kb, respectively. Heterokaryotic transformants showed two PCR bands (both of wild-type and knockout). Symbol star indicated deletion mutant. HDR, homology-directed repair.
