# Supplementary figures and images for: Upgrading of efficient and scalable CRISPR–Cas-mediated technology for genetic engineering in thermophilic fungus Myceliophthora thermophila
Source: Biotechnol Biofuels. 2019 Dec 23;12:293. doi: 10.1186/s13068-019-1637-y (PMC6927189; doi:10.1186/s13068-019-1637-y)

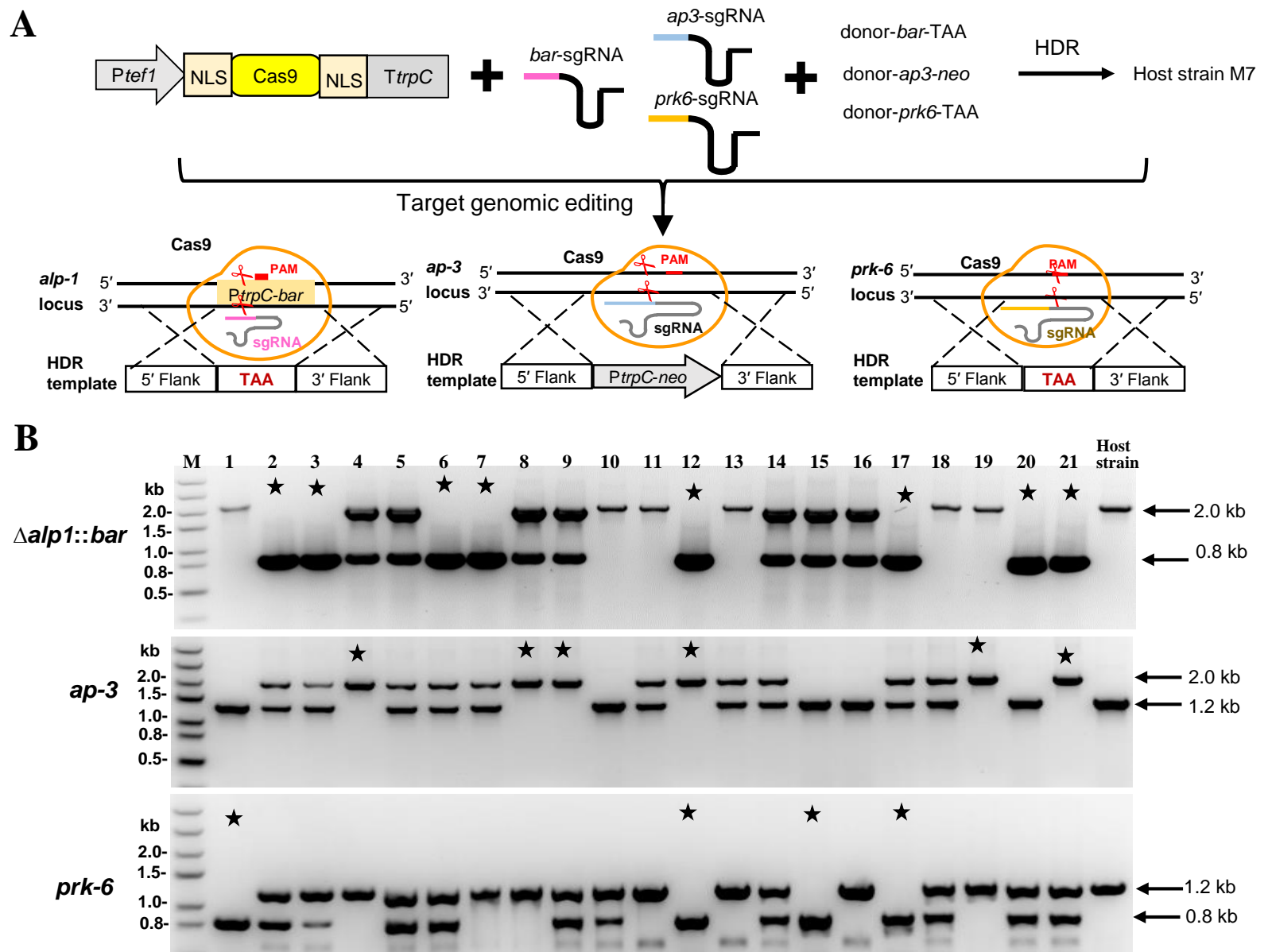

Supplement: Supplementary file 11 — Additional file 11: Figure S10. Third round of target genomic editing by CRISPR–Cas9 system. (A) Schematic of homologous recombination (HR) of bar, ap-3 and prk-6 mediated by Cas12a, array2 and donor DNA. (B) PCR analysis of triple-gene deletion of bar, ap-3 and prk-6 in selected 22 transformants using one primer (alp1-out-F2, ap3/prk6-out-F) located upstream of the 5′ flanking region of genomic DNA and the other primer (alp1-in-R2, gh1-1/res1-in-R) located in the 3′ flanking region of genomic DNA. The expected lengths of disrupted transformants of bar, ap3 and prk6 were 0.8, 2.0 and 0.8 kb, respectively, while those of the host strain (rightmost lane) was 2.0, 1.2 and 1.2 kb, respectively. Heterokaryotic transformants showed two PCR bands (both of wild-type and knockout). The symbol of star indicated deletion mutant. HDR, homology-directed repair. [file 13068_2019_1637_MOESM11_ESM.pdf]
