# Supplementary material for: Upgrading of efficient and scalable CRISPR–Cas-mediated technology for genetic engineering in thermophilic fungus Myceliophthora thermophila
Source: Biotechnol Biofuels. 2019 Dec 23;12:293. doi: 10.1186/s13068-019-1637-y (PMC6927189; doi:10.1186/s13068-019-1637-y)
Supplement: Supplementary file 12 — Additional file 12: Figure S11. SDS-PAGE of secreted protein from the eight mutant strains and wild type strain (WT) cultured for 6 days in Avicel inducing medium supplemented with 0.75% yeast extract. [file 13068_2019_1637_MOESM12_ESM.pdf]

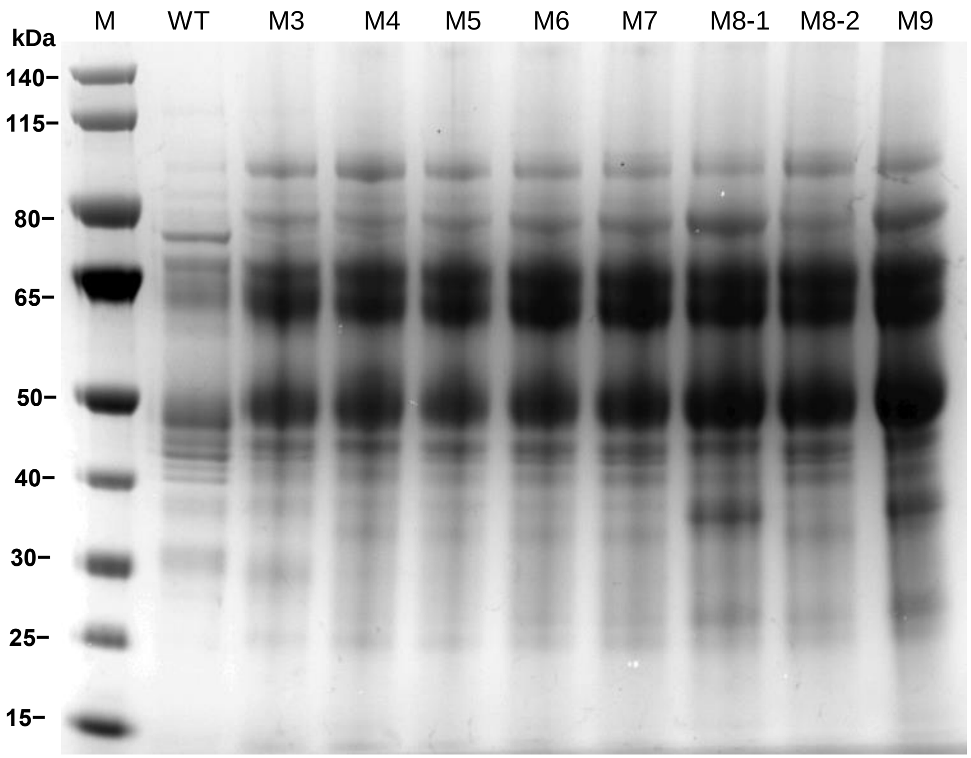

Figure S11. SDS-PAGE of secreted protein from the eight mutant strains and wild type strain (WT) cultured for 6 days in Avicel inducing medium supplemented with 0.75% yeast extract.
