# Supplementary material for: Upgrading of efficient and scalable CRISPR–Cas-mediated technology for genetic engineering in thermophilic fungus Myceliophthora thermophila
Source: Biotechnol Biofuels. 2019 Dec 23;12:293. doi: 10.1186/s13068-019-1637-y (PMC6927189; doi:10.1186/s13068-019-1637-y)
Supplement: Supplementary file 13 — Additional file 13: Table S2. List of Cas12a or Cas9 guide and PAM sequences in this study. [file 13068_2019_1637_MOESM13_ESM.docx]

**Additional file 13: Table S2 List of Cas12a or Cas9 guide and PAM sequences in this study.**

| **Target locus** | **Guide sequence** | **PAM** |
| --- | --- | --- |
| ***amdS*** | AGAGGCCGAACTGAAGATCACAG | TTTC |
| ***cre-1*** | GGCGGAAAGGGAGCAGACTCCAA | TTTC |
| ***res-1*** | TGCCTCGCCCCAGCTCCGGCCTG | TTTC |
| ***gh1-1*** | AGAACTACGCGCGCGTCATGTTC | TTTG |
| ***alp-1*** | CCGGCAAGCTCGAGGATGACGTC | TTTG |
| ***neo*** | TCAAGACCGACCTGTCCGGTGCC | TTTG |
| ***rca-1*** | ATCAGCAGCAACAATATCAGTAT | TTTG |
| ***hcr-1*** | CAGAGTCGGTACAAGTGCCCGAG | TTTC |
| ***bar*** | TGGCAGCTGGACTTCAGCCTGCC | TTTC |
| ***ap-3*** | GTCTCAGGCATGCGAACCTGCTC | TTTA |
| ***prk-6*** | AAACAAGACCCCAAGCCGAAGCA | TTTC |
| ***cre-1*** | GCAACGCGCAAAGTCTGCAG | TGG |
| ***res-1*** | GCCCTATGAGCCCTCGTACC | CGG |
| ***gh1-1*** | GACACATTCTGCGCCATCCC | CGG |
| ***alp-1*** | GTCTACCGCGGCAAGTTCAG | GGG |
| ***neo*** | GACTGGGCACAACAGACAAT | CGG |
| ***rca-1*** | GGACCTCTCGCCGTCGACGG | GGG |
| ***hcr-1*** | GACGTCGCCCTTTCCAGAGT | CGG |
| ***bar*** | GCAGGAACCGCAGGAGTGGA | CGG |
| ***ap-3*** | GATGCGCTCGAAGAATTCCT | CGG |
| ***prk-6*** | GCCATCGACATGTGGAGCGT | AGG |
