# Supplementary material for: Upgrading of efficient and scalable CRISPR–Cas-mediated technology for genetic engineering in thermophilic fungus Myceliophthora thermophila
Source: Biotechnol Biofuels. 2019 Dec 23;12:293. doi: 10.1186/s13068-019-1637-y (PMC6927189; doi:10.1186/s13068-019-1637-y)
Supplement: Supplementary file 14 — Additional file 14: Nucleotide sequence of the Cas12a expression cassette. Purple letters indicate the tef1 (translation elongation factor EF-1, MYCTH_2298136) promoter Ptef1. Blue letters indicate the nuclear localization signal. Red letters indicate the Cas12a gene. Gray letters indicate the TtrpC terminator from A. nidulans trpC gene. [file 13068_2019_1637_MOESM14_ESM.docx]

**Additional file 14:** Nucleotide sequence of the Cas12a expression cassette. Purple letters indicate the *tef1* (translation elongation factor EF-1, MYCTH_2298136) promoter P*tef1*. Blue letters indicate the nuclear localization signal. Red letters indicate the *Cas12a* gene. Gray letters indicate the *TtrpC* terminator from *A. nidulans* *trpC* gene.

>Cas12a expression cassette

TCCTCCGAGGTTCGACATCAGGGTTCGTCATAGGGAGTGAAACACCCGCCATGATTCCGTAGCCGCGCGCGAAGATACGAAGCAGATATTTCACGGACATGGCGGAGATACTTGTTTCCCGTACTAAGGTAGTCATGTCGGAGACATCTGAACGACAGAGCTGGCCAAGAGAACCGACCAGTTGCCCCAGGACGATCTAGACAAAAAAAAAGAGAGATGAGTGGGCCACTTTTGCCACAACATCGACGGCCCTGCGACCGCCCCCAGGCAAACAAACAAACCGCCGAACAATAATACTTTTGTCATTTTAGGAGGAGCGTTGTATGGATAAAAACAACATCTCGTTGCTGCAGAATGTGGACTTCAAACTTGCAGAAAATGGGAGGCGGATTTGCATGATCGGAGGGTAGTTGACTCACGCCGCAGGCTGCAAATCCGTCCTCCATTATTCCATGAACAACTTCGTAAGGTTGGGCTGAGCGCCAATGCCTAACGGACCGGGGGCCACAGCGCAACGTCCCACTTAAAGGCCAGCGTGACATGCCAGTTCCATACCAAGTAGTGGCACCAGAGGCGGCCAATGCTCAGTAAGGGCAGGGAGGGAGGCTCAAACGATTGGCAAAAAGAGGGGCTTGCCAGTTCAGTTCCCTGTGCGAGCGCGAGAGGGGCAGTTTCAAATCTGGAGGGGTGTGTTGCGCTGGTCTGAAGAGAAAGAGAAGACTGTACTTAATAATTGTTCAAAGAGTCCATCATCGCGTTGCGGACTCCTCTAGCTGTATTTAGAGCCCTATCATTACTTGTCGGGTGCGAATCAAAATACCGGGATGCAGCCCTCTGGCGATTTGCATGCGGTTGTGGAGGAAGTGAAGCCTGAATCGCGGGGCTGGGCGGCAAAGCACGACGTGAAATTCCTGGCGAAATTCGAGGGCTTGCCCCACCGTGGTTGAAGTTTTTGTGCTGCGTAACCCCACCAACCCGCCTTGCCCCTCCCGCCTGCCCATAAAAACTTCGACCCCTCCTCAAATCTTCTTCGATTCTTCCTCTTCACTTCCTTCGTCGGCATACCTGATTCAAGCAATCACCTGCCACTTTCAAGTGCGTATACCATCATCGATACACTGGTTCTTGACAAGTACATCGTCTCTAACTTTCCTTTTTGCAGTTTTCATTAAGCGCAAGTCGCCAGTTTCGTTCTTCAGAATGCCTCCAAGGAAACGCGCGAAGACCGAGGACGAGATGACCCAGTTCGAGGGGTTCACGAACCTGTATCAAGTGTCGAAGACGCTCCGGTTCGAGCTGATTCCGCAGGGCAAGACCCTCAAGCACATTCAGGAACAGGGCTTCATTGAGGAGGATAAGGCCCGCAACGATCACTACAAGGAATTAAAACCGATTATCGACCGCATTTACAAAACGTATGCGGATCAGTGCCTCCAACTCGTCCAGCTCGACTGGGAAAATCTGAGCGCCGCCATCGACAGCTATCGCAAAGAGAAGACCGAGGAGACGCGGAATGCGCTGATCGAAGAGCAAGCGACCTACCGCAATGCGATCCACGACTACTTTATCGGCCGCACGGATAACCTGACCGACGCGATTAACAAGCGCCACGCGGAAATCTACAAGGGGCTCTTCAAGGCGGAATTATTCAATGGGAAGGTGCTGAAGCAGCTGGGCACCGTCACGACGACGGAGCATGAGAATGCCCTGCTGCGCAGCTTCGACAAGTTCACCACGTACTTCTCGGGCTTCTACGAGAACCGCAAAAATGTCTTCTCGGCGGAAGACATCAGCACGGCGATCCCGCACCGCATTGTGCAAGATAATTTCCCCAAATTCAAGGAGAATTGTCACATCTTCACCCGCCTCATCACCGCCGTGCCCTCGCTGCGCGAGCATTTCGAGAACGTGAAGAAGGCGATCGGGATCTTCGTCAGCACGTCGATTGAAGAGGTGTTCAGCTTCCCCTTCTACAATCAGCTGCTGACGCAGACGCAAATTGACCTGTATAACCAACTGCTGGGGGGCATTTCGCGGGAGGCCGGCACCGAGAAAATTAAAGGCCTCAATGAAGTCCTGAACCTCGCGATCCAGAAGAATGACGAGACGGCCCATATCATCGCGTCGCTGCCGCACCGCTTCATTCCGCTGTTCAAGCAGATTCTCTCGGATCGGAATACCCTGAGCTTCATTCTCGAAGAGTTTAAGAGCGACGAAGAGGTCATTCAGTCGTTCTGCAAATACAAGACGCTCCTGCGGAACGAAAACGTCTTAGAAACCGCCGAAGCGCTGTTCAACGAGCTGAACAGCATCGATCTCACCCACATCTTCATCAGCCACAAGAAGTTAGAAACCATCAGCAGCGCGCTGTGCGACCACTGGGACACGCTCCGCAATGCGCTCTACGAGCGCCGGATCTCGGAGCTGACCGGGAAAATCACGAAGTCGGCCAAGGAGAAGGTCCAGCGGTCGCTCAAGCACGAGGACATCAACCTCCAGGAAATCATCTCGGCCGCGGGCAAGGAATTAAGCGAAGCCTTTAAGCAGAAGACCAGCGAAATCCTCAGCCACGCCCATGCCGCCCTCGACCAACCCCTCCCCACCACCCTCAAGAAGCAGGAAGAGAAGGAAATCCTGAAGTCGCAACTCGACAGCCTCCTGGGCCTGTACCACCTCCTGGACTGGTTCGCGGTCGATGAATCGAACGAGGTCGATCCCGAGTTCAGCGCGCGGCTCACCGGGATCAAGCTGGAAATGGAACCCAGCCTGAGCTTCTACAACAAGGCGCGCAATTACGCCACGAAGAAGCCGTATTCGGTGGAGAAGTTTAAGCTGAATTTCCAAATGCCGACGCTGGCCTCGGGGTGGGACGTCAACAAAGAGAAGAACAATGGGGCCATCCTGTTCGTCAAGAACGGCCTGTACTACCTCGGCATTATGCCCAAACAGAAGGGCCGCTATAAGGCGCTGTCGTTCGAGCCGACCGAGAAGACGAGCGAGGGGTTTGACAAAATGTATTATGACTACTTCCCGGATGCGGCCAAGATGATCCCCAAGTGCAGCACGCAACTCAAAGCCGTGACGGCCCACTTCCAAACGCACACCACCCCCATCCTCCTCAGCAACAACTTCATCGAGCCGTTAGAAATCACGAAGGAAATTTATGACCTGAACAACCCCGAGAAAGAGCCGAAGAAGTTCCAGACCGCCTATGCGAAAAAGACCGGCGACCAGAAGGGGTATCGCGAGGCGCTCTGCAAGTGGATCGACTTTACCCGCGACTTCCTCTCGAAGTATACGAAGACGACCAGCATCGACTTATCTTCTCTGCGGCCCAGCAGCCAATACAAGGATTTAGGCGAGTACTACGCCGAGCTGAACCCCCTCCTCTACCACATTAGCTTCCAGCGGATTGCCGAGAAAGAGATCATGGATGCCGTGGAAACGGGCAAGCTCTACCTCTTCCAAATTTATAACAAAGACTTTGCCAAGGGGCACCATGGGAAACCCAACCTGCACACGCTGTACTGGACCGGCCTGTTTAGCCCGGAGAACCTGGCCAAGACCAGCATCAAGCTCAACGGCCAGGCCGAATTATTTTATCGGCCGAAGTCGCGGATGAAACGGATGGCGCACCGGCTGGGCGAAAAGATGCTGAATAAGAAGCTGAAGGATCAAAAGACGCCCATCCCCGATACGCTGTACCAGGAACTGTATGACTACGTCAACCACCGCCTCTCGCACGATCTCAGCGATGAGGCGCGGGCCCTGCTCCCCAATGTCATCACCAAGGAAGTGAGCCACGAGATTATCAAGGACCGCCGGTTCACGAGCGACAAGTTTTTCTTTCACGTCCCGATTACCCTCAACTATCAGGCCGCGAATAGCCCGAGCAAGTTCAACCAACGGGTGAATGCGTATCTCAAGGAGCACCCGGAAACCCCCATCATCGGGATCGACCGCGGCGAGCGCAACCTCATCTATATCACGGTCATCGACTCGACCGGGAAGATCCTGGAGCAACGCAGCCTGAATACGATCCAGCAGTTTGACTATCAGAAGAAGCTCGATAATCGCGAAAAGGAACGGGTGGCCGCGCGGCAGGCCTGGAGCGTGGTCGGGACGATCAAGGATCTCAAGCAAGGCTACCTCAGCCAGGTCATCCACGAGATTGTGGATCTCATGATCCACTACCAGGCCGTGGTCGTCCTCGAAAACCTCAATTTCGGCTTTAAGTCGAAACGCACCGGCATCGCCGAAAAAGCGGTGTATCAACAGTTCGAGAAGATGCTGATTGACAAGCTGAACTGCCTCGTCCTGAAAGACTATCCCGCCGAAAAGGTGGGCGGGGTCCTCAACCCCTACCAGCTCACGGATCAGTTCACCTCGTTCGCCAAAATGGGGACGCAGTCGGGCTTCCTCTTCTACGTGCCGGCCCCGTACACGTCGAAGATCGATCCCCTGACCGGCTTCGTGGACCCCTTCGTGTGGAAGACCATCAAGAATCACGAGTCGCGGAAGCACTTTCTGGAGGGGTTCGACTTCCTCCACTATGACGTCAAGACGGGGGACTTCATCCTCCACTTCAAGATGAATCGGAATCTGTCGTTCCAACGGGGCCTGCCGGGCTTCATGCCCGCCTGGGACATTGTCTTCGAGAAGAACGAGACGCAATTCGATGCGAAGGGCACCCCGTTCATCGCCGGGAAGCGGATTGTCCCCGTGATCGAGAACCATCGGTTCACCGGGCGGTATCGCGATCTCTACCCCGCCAACGAGCTGATCGCCCTGTTAGAAGAAAAAGGCATCGTGTTCCGCGACGGGAGCAACATTCTGCCCAAGCTCTTAGAAAACGACGATAGCCATGCCATCGATACGATGGTGGCCCTCATTCGGTCGGTGCTCCAAATGCGGAATAGCAATGCGGCGACGGGCGAGGACTACATCAACTCGCCCGTGCGGGATCTCAACGGCGTGTGCTTCGATAGCCGCTTTCAGAACCCCGAGTGGCCGATGGATGCGGACGCCAACGGCGCCTATCACATTGCCCTGAAGGGCCAGCTGCTCCTCAATCACCTGAAGGAGAGCAAGGACCTCAAGCTCCAGAACGGCATCTCGAACCAAGACTGGCTCGCCTATATTCAGGAGCTGCGCAATGACCCCAAGAAGAAACGCAAGGTTGATCCGAAGAAGAAGCGGAAGGTTCCTCCTCCGAGGAAACGTGCCAAAACAGAAGATGAGTGATGAGGATCCACTTAACGTTACTGAAATCATCAAACAGCTTGACGAATCTGGATATAAGATCGTTGGTGTCGATGTCAGCTCCGGAGTTGAGACAAATGGTGTTCAGGATCTCGATAAGATACGTTCATTTGTCCAAGCAGCAAAGAGTGCCTTCTAGTGATTTAATAGCTCCATGTCAACAAGAATAAAACGCGTTTTCGGGTTTACCTCTTCCAGATACAGCTCATCTGCAATGCATTAATGCATTGACTGCAACCTAGTAACGCCTTACAGGCTCCGGCGAAGAGAAGAATAGCTTAGCAGAGCTATTTTCATTTTCGGGAGACGAGATCAAGCAGATCAACGGTCGTCAAGAGACCTACGAGACTGAGGAATCCGCTCTTGGCTCCACGCGACTATATATTTGTCTCTAATTGTACTTTGACATGCTCCTCTTCTTTACTCTGATAGCTTGACTATGAAAATTCCGTCACCAGCTCCTGGGTTCGCAAAGATAATTGCATGTTTCTTCCTTGAACTCTCAAGCCTACAGGACACACATTCATCGTAGGTATAAACCTCGAAATCATTTCCTACTAAGATGGTATACAATAGTAACCATGCATGGTTGCCTAGTGAATGCTCCGTAACACCCAATACGCCGGCCGAAACTTTTTTACAACTCTCCTATGAGTCGTTTACCCAGAATGCACAGGTACACTTGTTTAGAGG
