# Supplementary material for: Upgrading of efficient and scalable CRISPR–Cas-mediated technology for genetic engineering in thermophilic fungus Myceliophthora thermophila
Source: Biotechnol Biofuels. 2019 Dec 23;12:293. doi: 10.1186/s13068-019-1637-y (PMC6927189; doi:10.1186/s13068-019-1637-y)
Supplement: Supplementary file 15 — Additional file 15. Nucleotide sequence of the crRNA expression cassettes. Blue letters indicate the RNA polymerase III U6 snRNA promoter. Red letters indicate the 19 nt direct repeat. Green letters indicate the target sequence of amdS, cre-1, res-1, gh1-1, alp-1, neo, rca-1, hcr-1, bar, ap-3, or prk-6, respectively. [file 13068_2019_1637_MOESM15_ESM.docx]

**Additional file 15:** Nucleotide sequence of the crRNA expression cassettes. Blue letters indicate the RNA polymerase III U6 snRNA promoter. Red letters indicate the 19 nt direct repeat. Green letters indicate the target sequence of *amdS*, *cre-1*, *res-1 gh1-1*, *alp-1*, *neo*, *rca-1*, *hcr-1*, *bar*, *ap-3*, or *prk-6*, respectively.

>RNA polymerase III U6 snRNA promoter

AGGATCGGTGGAGTGAAGTTCGGAATCGAGGTTCGGCGATGGGTCGTAAGCATGGCGACTTCGAACTTACTTGCACTGGCAAGCGTTGCCAGAACGGCGAGAAAAAGAAGGGTAAGCGATATTCGCGTCATGATGGACTGTTCCTTTTGGAACAGTAGTTGTTGTGGGAAGACTATGTCACACTTGCCCACCTGCAAGGCCAGGGTCGTGGTCGAACGAGACCAGCCTCGGCGCTGCTGGGAGCTCAAGATGGGCACGTTTGATTCGTTAGACGTCAACAAGGCTGGAGTTCCTAGTGACAGCCAAAGGCACAGCCACATTAAGTGGCGCTTTATCTGTCCACTAAGGTTCAATTGTGGCTTTGAGCCGCGCAGTGTGCAGTCGTGCATTGGCCACCTAGCTAGCAGTATTTAAGATCCTCTTCTCTCCCGAGATCTTCCTCCTCTTCTTTTCTTTCTTTCCTCG

>crRNA**-***amdS* expression cassette with U6 promoter

AGGATCGGTGGAGTGAAGTTCGGAATCGAGGTTCGGCGATGGGTCGTAAGCATGGCGACTTCGAACTTACTTGCACTGGCAAGCGTTGCCAGAACGGCGAGAAAAAGAAGGGTAAGCGATATTCGCGTCATGATGGACTGTTCCTTTTGGAACAGTAGTTGTTGTGGGAAGACTATGTCACACTTGCCCACCTGCAAGGCCAGGGTCGTGGTCGAACGAGACCAGCCTCGGCGCTGCTGGGAGCTCAAGATGGGCACGTTTGATTCGTTAGACGTCAACAAGGCTGGAGTTCCTAGTGACAGCCAAAGGCACAGCCACATTAAGTGGCGCTTTATCTGTCCACTAAGGTTCAATTGTGGCTTTGAGCCGCGCAGTGTGCAGTCGTGCATTGGCCACCTAGCTAGCAGTATTTAAGATCCTCTTCTCTCCCGAGATCTTCCTCCTCTTCTTTTCTTTCTTTCCTCGAATTTCTACTCTTGTAGATAGAGGCCGAACTGAAGATCACAGTTTTTT

>sgRNA**-***cre-1* expression cassette with U6 promoter

AGGATCGGTGGAGTGAAGTTCGGAATCGAGGTTCGGCGATGGGTCGTAAGCATGGCGACTTCGAACTTACTTGCACTGGCAAGCGTTGCCAGAACGGCGAGAAAAAGAAGGGTAAGCGATATTCGCGTCATGATGGACTGTTCCTTTTGGAACAGTAGTTGTTGTGGGAAGACTATGTCACACTTGCCCACCTGCAAGGCCAGGGTCGTGGTCGAACGAGACCAGCCTCGGCGCTGCTGGGAGCTCAAGATGGGCACGTTTGATTCGTTAGACGTCAACAAGGCTGGAGTTCCTAGTGACAGCCAAAGGCACAGCCACATTAAGTGGCGCTTTATCTGTCCACTAAGGTTCAATTGTGGCTTTGAGCCGCGCAGTGTGCAGTCGTGCATTGGCCACCTAGCTAGCAGTATTTAAGATCCTCTTCTCTCCCGAGATCTTCCTCCTCTTCTTTTCTTTCTTTCCTCGAATTTCTACTCTTGTAGATGGCGGAAAGGGAGCAGACTCCAATTTTTT

>crRNA**-***res-1* expression cassette with U6 promoter

AGGATCGGTGGAGTGAAGTTCGGAATCGAGGTTCGGCGATGGGTCGTAAGCATGGCGACTTCGAACTTACTTGCACTGGCAAGCGTTGCCAGAACGGCGAGAAAAAGAAGGGTAAGCGATATTCGCGTCATGATGGACTGTTCCTTTTGGAACAGTAGTTGTTGTGGGAAGACTATGTCACACTTGCCCACCTGCAAGGCCAGGGTCGTGGTCGAACGAGACCAGCCTCGGCGCTGCTGGGAGCTCAAGATGGGCACGTTTGATTCGTTAGACGTCAACAAGGCTGGAGTTCCTAGTGACAGCCAAAGGCACAGCCACATTAAGTGGCGCTTTATCTGTCCACTAAGGTTCAATTGTGGCTTTGAGCCGCGCAGTGTGCAGTCGTGCATTGGCCACCTAGCTAGCAGTATTTAAGATCCTCTTCTCTCCCGAGATCTTCCTCCTCTTCTTTTCTTTCTTTCCTCGAATTTCTACTCTTGTAGATTGCCTCGCCCCAGCTCCGGCCTGTTTTTT

>crRNA**-***gh1-1* expression cassette with U6 promoter

AGGATCGGTGGAGTGAAGTTCGGAATCGAGGTTCGGCGATGGGTCGTAAGCATGGCGACTTCGAACTTACTTGCACTGGCAAGCGTTGCCAGAACGGCGAGAAAAAGAAGGGTAAGCGATATTCGCGTCATGATGGACTGTTCCTTTTGGAACAGTAGTTGTTGTGGGAAGACTATGTCACACTTGCCCACCTGCAAGGCCAGGGTCGTGGTCGAACGAGACCAGCCTCGGCGCTGCTGGGAGCTCAAGATGGGCACGTTTGATTCGTTAGACGTCAACAAGGCTGGAGTTCCTAGTGACAGCCAAAGGCACAGCCACATTAAGTGGCGCTTTATCTGTCCACTAAGGTTCAATTGTGGCTTTGAGCCGCGCAGTGTGCAGTCGTGCATTGGCCACCTAGCTAGCAGTATTTAAGATCCTCTTCTCTCCCGAGATCTTCCTCCTCTTCTTTTCTTTCTTTCCTCGAATTTCTACTCTTGTAGATAGAACTACGCGCGCGTCATGTTCTTTTTT

>crRNA Array1 expression cassette with U6 promoter

AGGATCGGTGGAGTGAAGTTCGGAATCGAGGTTCGGCGATGGGTCGTAAGCATGGCGACTTCGAACTTACTTGCACTGGCAAGCGTTGCCAGAACGGCGAGAAAAAGAAGGGTAAGCGATATTCGCGTCATGATGGACTGTTCCTTTTGGAACAGTAGTTGTTGTGGGAAGACTATGTCACACTTGCCCACCTGCAAGGCCAGGGTCGTGGTCGAACGAGACCAGCCTCGGCGCTGCTGGGAGCTCAAGATGGGCACGTTTGATTCGTTAGACGTCAACAAGGCTGGAGTTCCTAGTGACAGCCAAAGGCACAGCCACATTAAGTGGCGCTTTATCTGTCCACTAAGGTTCAATTGTGGCTTTGAGCCGCGCAGTGTGCAGTCGTGCATTGGCCACCTAGCTAGCAGTATTTAAGATCCTCTTCTCTCCCGAGATCTTCCTCCTCTTCTTTTCTTTCTTTCCTCGAATTTCTACTCTTGTAGATGGCGGAAAGGGAGCAGACTCCAAAATTTCTACTCTTGTAGATTGCCTCGCCCCAGCTCCGGCCTGAATTTCTACTCTTGTAGATAGAACTACGCGCGCGTCATGTTCTTTTTT

>crRNA Array2 expression cassette with U6 promoter

AGGATCGGTGGAGTGAAGTTCGGAATCGAGGTTCGGCGATGGGTCGTAAGCATGGCGACTTCGAACTTACTTGCACTGGCAAGCGTTGCCAGAACGGCGAGAAAAAGAAGGGTAAGCGATATTCGCGTCATGATGGACTGTTCCTTTTGGAACAGTAGTTGTTGTGGGAAGACTATGTCACACTTGCCCACCTGCAAGGCCAGGGTCGTGGTCGAACGAGACCAGCCTCGGCGCTGCTGGGAGCTCAAGATGGGCACGTTTGATTCGTTAGACGTCAACAAGGCTGGAGTTCCTAGTGACAGCCAAAGGCACAGCCACATTAAGTGGCGCTTTATCTGTCCACTAAGGTTCAATTGTGGCTTTGAGCCGCGCAGTGTGCAGTCGTGCATTGGCCACCTAGCTAGCAGTATTTAAGATCCTCTTCTCTCCCGAGATCTTCCTCCTCTTCTTTTCTTTCTTTCCTCATTTCTACTCTTGTAGATTCAAGACCGACCTGTCCGGTGCCAATTTCTACTCTTGTAGATCCGGCAAGCTCGAGGATGACGTCAATTTCTACTCTTGTAGATATCAGCAGCAACAATATCAGTATAATTTCTACTCTTGTAGATCAGAGTCGGTACAAGTGCCCGAGTTTTTT

>crRNA Array3 expression cassette with U6 promoter

AGGATCGGTGGAGTGAAGTTCGGAATCGAGGTTCGGCGATGGGTCGTAAGCATGGCGACTTCGAACTTACTTGCACTGGCAAGCGTTGCCAGAACGGCGAGAAAAAGAAGGGTAAGCGATATTCGCGTCATGATGGACTGTTCCTTTTGGAACAGTAGTTGTTGTGGGAAGACTATGTCACACTTGCCCACCTGCAAGGCCAGGGTCGTGGTCGAACGAGACCAGCCTCGGCGCTGCTGGGAGCTCAAGATGGGCACGTTTGATTCGTTAGACGTCAACAAGGCTGGAGTTCCTAGTGACAGCCAAAGGCACAGCCACATTAAGTGGCGCTTTATCTGTCCACTAAGGTTCAATTGTGGCTTTGAGCCGCGCAGTGTGCAGTCGTGCATTGGCCACCTAGCTAGCAGTATTTAAGATCCTCTTCTCTCCCGAGATCTTCCTCCTCTTCTTTTCTTTCTTTCCTCATTTCTACTCTTGTAGATTGGCAGCTGGACTTCAGCCTGCCAATTTCTACTCTTGTAGATGTCTCAGGCATGCGAACCTGCTCAATTTCTACTCTTGTAGATAAACAAGACCCCAAGCCGAAGCATTTTTT
